# A high-risk, Double-Hit, group of newly diagnosed myeloma identified by genomic analysis

**DOI:** 10.1038/s41375-018-0196-8

**Published:** 2018-07-02

**Authors:** Brian A. Walker, Konstantinos Mavrommatis, Christopher P. Wardell, T. Cody Ashby, Michael Bauer, Faith Davies, Adam Rosenthal, Hongwei Wang, Pingping Qu, Antje Hoering, Mehmet Samur, Fadi Towfic, Maria Ortiz, Erin Flynt, Zhinuan Yu, Zhihong Yang, Dan Rozelle, John Obenauer, Matthew Trotter, Daniel Auclair, Jonathan Keats, Niccolo Bolli, Mariateresa Fulciniti, Raphael Szalat, Phillipe Moreau, Brian Durie, A. Keith Stewart, Hartmut Goldschmidt, Marc S. Raab, Hermann Einsele, Pieter Sonneveld, Jesus San Miguel, Sagar Lonial, Graham H. Jackson, Kenneth C. Anderson, Herve Avet-Loiseau, Nikhil Munshi, Anjan Thakurta, Gareth Morgan

**Affiliations:** 1 Myeloma Institute, University of Arkansas for Medical Sciences, Little Rock, AR USA; 2 Celgene Corporation, San Francisco, CA USA; 3 Cancer Research and Biostatistics, Seattle, WA USA; 4 Dana-Farber Cancer Institute, Harvard Medical School, Boston, MA USA; 5 Celgene Corporation, Summit, NJ USA; 6 Celgene Institute of Translational Research Europe, Sevilla, Spain; 7 Rancho BioSciences, San Diego, CA USA; 8 Multiple Myeloma Research Foundation, Norwalk, CT USA; 9 Translational Genomics Research Institute, Phoenix, AZ USA; 10 University of Milan, Milano, Italy; 11 University of Nantes, Nantes, France; 12 Cedars-Sinai Samuel Oschin Cancer Center, Los Angeles, CA USA; 13 Department of Hematology, Mayo Clinic, Scottsdale, AZ USA; 14 Department of Medicine V, Hematology and Oncology, University Hospital of Heidelberg, Heidelberg, Germany; 15 German Cancer Research Center (DKFZ), Heidelberg, Heidelberg, Germany; 16 Department of Internal Medicine II, Wurzburg University, Wurzburg, Germany; 17 Department of Hematology, Erasmus MC Cancer Institute, Rotterdam, The Netherlands; 18 Clinica Universidad de Navarra, Centro Investigacion Medica Aplicada (CIMA), IDISNA, CIBERONC, Pamplona, Spain; 19 Winship Cancer Institute, Emory University, Atlanta, GA USA; 20 Department of Haematology, Newcastle University, Newcastle, UK; 21 Centre de Recherche en Cancérologie de Toulouse Institut National de la Santé et de la Recherche Médicale, U1037 Toulouse, France; 22 L’Institut Universitaire du Cancer de Toulouse Oncopole, Centre Hospitalier Universitaire, Toulouse, France

**Keywords:** Risk factors, Cancer genomics

## Abstract

Patients with newly diagnosed multiple myeloma (NDMM) with high-risk disease are in need of new treatment strategies to improve the outcomes. Multiple clinical, cytogenetic, or gene expression features have been used to identify high-risk patients, each of which has significant weaknesses. Inclusion of molecular features into risk stratification could resolve the current challenges. In a genome-wide analysis of the largest set of molecular and clinical data established to date from NDMM, as part of the Myeloma Genome Project, we have defined DNA drivers of aggressive clinical behavior. Whole-genome and exome data from 1273 NDMM patients identified genetic factors that contribute significantly to progression free survival (PFS) and overall survival (OS) (cumulative *R*^2^ = 18.4% and 25.2%, respectively). Integrating DNA drivers and clinical data into a Cox model using 784 patients with ISS, age, PFS, OS, and genomic data, the model has a cumlative R^2^ of 34.3% for PFS and 46.5% for OS. A high-risk subgroup was defined by recursive partitioning using either a) bi-allelic *TP53* inactivation or b) amplification (≥4 copies) of *CKS1B* (1q21) on the background of International Staging System III, comprising 6.1% of the population (median PFS = 15.4 months; OS = 20.7 months) that was validated in an independent dataset. Double-Hit patients have a dire prognosis despite modern therapies and should be considered for novel therapeutic approaches.

## Introduction

We have made consistent therapeutic progress for patients with newly diagnosed multiple myeloma (NDMM) over the last two decades; however, not all patients, especially high-risk patients, have uniformly derived the benefit [1–12]. Patients with high-risk disease are associated with a poor prognosis, but identifying these patients at diagnosis remain a challenge. Multiple definitions of high-risk have evolved over time, but today no definition is uniformly accepted or implemented in clinical practice. Current approaches rely upon cytogenetic and clinical biomarkers to define high-risk, including the International Staging System (ISS) group III, the presence of adverse translocations, and 17p deletion (del17p); [3, 13, 14] however, non-uniform application and interpretation of these variables have resulted in the description of different high-risk groups with varying outcomes. For example, the high-risk group identified by the ISS is 33.6% with a median overall survival (OS) of 29 months, while the International Myeloma Working Group (IMWG) identified a high-risk group of 20% with a 4-year progression free survival (PFS) of 12% and OS of 35% [3, 13]. The revised ISS (R-ISS) is the most recent risk stratification approach and incorporates the genetic markers t(4;14) and del17p, but not 1q gain or mutational data from *TP53* as the data were not available [14]. The high-risk group in the R-ISS classification comprised 10% and had a median PFS of 29 months and 5-year OS of 40%. Thus, although high-risk groups can be identified, the definition of these groups and the specificity for very poor outcome varies, confounding effective clinical decision making and the design of risk-adjusted trials.

DNA drivers of biological activity are key determinants of cancer behavior, are robust, and easily measured in the clinical laboratory. While a number of mutational markers have been identified [5, 15] as being associated with prognosis, there has been no comprehensive approach to integrate such markers into risk stratification approaches because of small study size, use of focused disease panels, and lack of follow-up, all of which can confound interpretation of the results [2, 5, 15, 16]. As part of the Myeloma Genome Project (MGP), we have established the largest repository of uniformly called molecular data associated with clinical outcomes in NDMM, providing adequate power to identify the variables associated with very high-risk groups. Next-generation sequencing (NGS) data was analyzed to comprehensively provide genome-wide information on structural, mutational, and copy number (CN) drivers, which can provide a global view of association with outcome. We focused the analysis on the key clinical problem of identifying high-risk patients destined for early relapse and death, where a change in treatment strategy could result in improved outcome.

The analysis identified a previously undescribed high-risk segment that is defined by two DNA-based genomic markers and key clinical data, that we refer to as Double-Hit MM. Double-Hit patients have extremely poor prognosis, even compared to other definitions of high risk. We propose that future baseline risk assessments should include screening for these patients to provide opportunities for risk-adjusted therapy.

## Materials and methods

### Patient characteristics and statistical analysis

MGP is an ongoing collaborative research initiative to assemble and uniformly analyze genetic datasets that have been generated on samples obtained from patients with NDMM (*n* = 1273). The project has collected multilevel genetic data, giving it the power to identify the full spectrum of molecular drivers. The data has been assembled from multiple groups across Europe and the USA, and has undergone extensive quality control.

NGS data were processed and analyzed uniformly, Supplementary Methods, Supplementary Figure 3, Supplementary Table 1, and associated genomics results [17]. For prognostic analyses, patients aged ≥75 years were excluded because their prognosis was poor irrespective of the genetic background, leaving 863 patients. A final set of 784 patients with a complete dataset comprising variant and CN calls, survival data and ISS had a median follow-up of 22.9 months (range 0.0–52.9), median PFS of 31.2 months (range 0.0–51.6), and median OS not reached, Supplementary Table 1. Of note, lactate dehydrogenase (LDH) values were not universally available, preventing the calculation of R-ISS for all patients, hence, the IMWG risk criteria were used [13]. The final analysis subset (*n* = 784) compares similarly to the full dataset (*n* = 1273) for major clinical features other than age due to the exclusion of patients ≥75 years, Supplementary Table 1. DNA-driver status was assigned based on previously established criteria, as outlined in the accompanying genomic results [17–19]. The study had 95% power to identify the significantly mutated genes occurring at the 5% level, accompanying genomic results [17].

Detailed statistical methods are available in the Supplementary Methods. The Kaplan–Meier estimator was used to calculate time-to-event distributions. Stepwise Cox regression was used to select variables and estimate the effects of significant covariates for time-to-event outcomes. Cumulative *R*^2^, the percentage of variance explained by a factor or set of factors, was calculated [20] for the factors entering regression models based on the order in which they were entered into the models. Recursive partitioning was used to classify patients into risk strata. Genetic factors significantly associated with PFS in the multivariate Cox model were considered first, followed by the addition of age and ISS in the presence of these adverse genetic factors.

## Results

### Genetic contributors to outcome

**Fig. 1:**
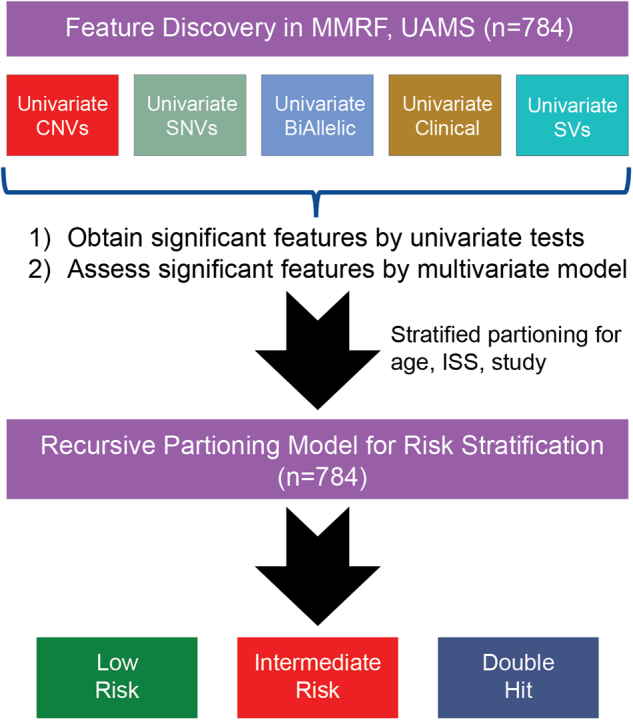
Study outline. Data from 784 patients were used to identify univariate and multivariate features associated with PFS. Genetic features were used for multivariate modeling and were also used in a recursive partitioning model with significantly different outcomes

The study outline is shown in Fig. 1. A summary of baseline clinical and molecular features is provided, Supplementary Table 1. The dataset used to establish the risk models has 27% ISS Stage III patients, comparable to several recent studies [7, 21]. A key distinction between this population (*n* = 784) and other datasets is that patients ≥75 years were removed (Supplementary Figure 3). The study group comprised 45% of patients aged 65–74 years, a greater percentage than other similar studies [14]. The impacts of cytogenetic variables, immunoglobulin heavy chain gene (*IGH*) translocations, and hyperdiploidy on outcome are provided in Supplementary Figure 4. The presence of *IGH* translocations including t(4;14) was significantly associated with worse PFS but not OS, while hyperdiploidy was not associated with outcome.

**Fig. 2:**
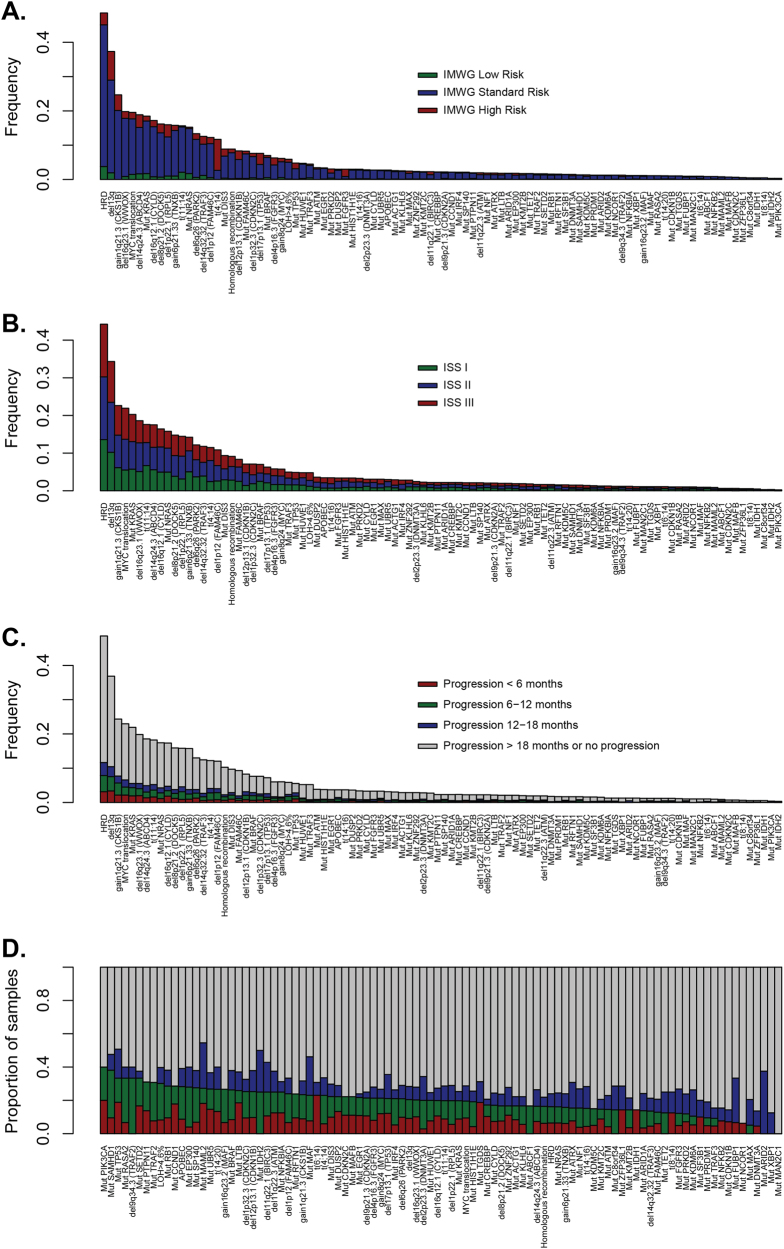
The association of myeloma-acquired genetic variants with clinical risk groups. **a** The distribution of driver mutations, translocations, and copy number alterations by IMWG risk status. It can be seen that a limited number of variables explain a proportion of risk, as would be anticipated based on how the IMWG risk status is assessed, but it can be seen clearly that these variants do not explain a significant amount of variability in clinical outcome. **b** The distribution of driver mutations, translocations, and copy number alterations by ISS. The distribution shows the independence of ISS from the genetic data, suggesting that a patient’s ISS stage cannot be predicted by mutational diagnosis (and vice-versa); also, that using both could be important for modeling patient outcomes. **c** Bar plot shows the contribution of each driver variant to relapse, with a breakdown of PFS over <6 months/6–12 months/12–18 months/>18 months, or no progression. Patients with censored follow-up <18 months were excluded from the analysis. **d** The same data as in plot (**c**) was only expressed as a proportion, with features sorted by the proportion, of patients who relapsed within the first year of therapy. Differences in rates in early relapse across genetic features suggest a motivation for the inclusion of such features in predictive modeling for poor patient outcome

Structural rearrangements, SNV mutations, and CN abnormalities were called to identify key MM driver genes [17]. To determine if these variables contribute to the risk status, we plotted them against currently used markers of risk, including ISS, IMWG, and time to relapse (Fig. 2). The distribution of genetic features was not dependent upon IMWG risk group, except for t(4;14) and del17p, which are part of the definition of risk (Fig. 2a), or ISS (Fig. 2b). This indicates that clinical and genetic features are not strongly associated, and clinical prognostic models may benefit from the inclusion of genetic factors. The distribution of genetic features by time to relapse (Fig. 2c), and the percentage breakdown of timing of relapse within each genetic feature is shown in Fig. 2d. The key differences in the percentage of patients with early relapse defined by genetic markers provide a rationale for building the models to predict the poor outcome, featuring genetic markers as covariates.

### Univariate Cox regression

**Fig. 3:**
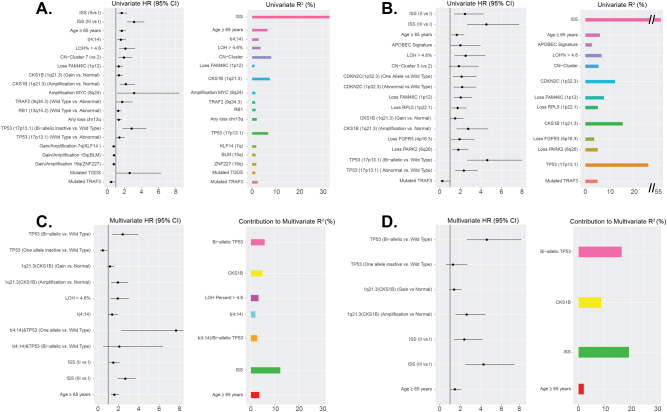
Molecular and clinical features associated with outcome. Significant associations of genetic and clinical factors with PFS (**a**) and OS (**b**) in univariate analyses. Covariates investigated include, age, ISS, *IGH* translocations, *MYC* translocation, APOBEC signature, hyperdiploidy, LOH%, homologous recombination deficiency mutations, copy number cluster, mutational data, copy number data, and bi-allelic inactivation data. Covariates significantly associated with at least one of PFS or OS (Wald *P* ≤ 0.05) in univariate models are presented. **c** The final multivariate model for PFS containing clinical and genetic factors has a cumulative R-squared of 34.3% compared to a cumulative R-squared of 18.4% for the model developed containing only genetic factors. **d** The final model for OS contains clinical and genetic factors, and has a cumulative R-squared of 46.5% compared to a cumulative R-squared of 25.2% for the model developed containing only genetic factors

To determine the markers which contribute to high-risk disease, we analyzed 784 patients with complete ISS, PFS, OS, and genomic data. The results of univariate analyses for PFS and OS for molecular features are shown, Supplementary Tables 2–5. All features significantly associated with either PFS or OS in a univariate model are presented in Fig. 3a, b and Supplementary Tables 7 and 8. For PFS, a protective effect was associated with ISS I, mutation of *TRAF3*, and gain/amplification of 7q, 15q, and 19q. An adverse association was seen for ISS II/III, age ≥ 65 years, t(4;14), mutation of *TGDS*, gain or amplification of *CKS1B* or *MYC*, loss of *FAM46C*, and mono-allelic or bi-allelic inactivation of *RB1*, *TRAF2*, or *TP53*. Additionally, the level of global loss of heterozygosity (LOH) > 4.6% and CN clusters associated with 1q gain/amp (described in associated genomics results) [17] were associated with poor PFS (Supplementary Methods). Similarly for OS (Fig. 3b), ISS stage I and mutation of *TRAF3* were associated with a positive effect, whereas an adverse association was seen with ISS stage II/III, age ≥ 65 years, the APOBEC signature, global LOH > 4.6%, mutation *TGDS*, gain or amplification of *CKS1B*, loss of *FAM46C*, *RPL5, FGFR3*, or *PARK2*, and mono-allelic or bi-allelic inactivation of *CDNK2C* and *TP53*. Although loss or mutation of *TP53* was significantly associated with shorter OS, the effect was strongest with bi-allelic inactivation. The opposite was seen with *FAM46C*, where loss was significant for the outcome, but mutation was not (Supplementary Figure 5). The combined effect of bi-allelic loss of *FAM46C* was not significant, whereas loss alone was, Supplementary Tables 2 and 4.

Interactions between molecular variables were tested, Supplementary Table 6. For PFS, a significant interaction was observed univariately between t(4;14) and bi-allelic *TP53* (*P* = 0.0217), and between *ZNF426* (chr. 19) gain and *FAM46C* loss (*P* = 0.0202), while the interaction between *CKS1B* gain/amplification and *ABCD4* (chr. 14) loss was just above the threshold for significance (*P* = 0.0538). For OS, a significant interaction was observed between *CKS1B* [1q] gain/amplification and *FAM46C* [1p] loss (*P* = 0.0461), Supplementary Figure 6 and Supplementary Table 6. In all cases, the presence of both features was associated with poor prognosis.

### Multivariate Cox regression

The approach for multivariate Cox modeling was to (1) obtain a model by allowing entry of only genetic features, (2) include all significant interactions after testing all possible pairs of interactions among significant genetic factors selected by multivariate Cox regression, and finally (3) adjust this model for age, ISS, and study site to obtain a final composite model for outcome. The initial PFS model for genetic markers (prior to adjustment for clinical factors) featured: *CKS1B* gain/amplification (three-level variable), bi-allelic *TP53* inactivation (three-level variable), LOH > 4.6%, and t(4;14) (Fig. 3c and Supplementary Table 9). Pair-wise interactions between these CN and bi-allelic inactivated covariates were tested for significance, as were the significant and borderline-significant interactions in the univariate setting; ultimately, only t(4;14) and bi-allelic *TP53* interactions entered into the model as significant in multivariate analysis. After adjustment for age, ISS, and study site, all aforementioned genetic factors remained in the model, but study site indicator was removed from the final model due to non-significance.

The initial model for OS based on genetic markers (prior to adjustment for clinical factors) featured: bi-allelic *TP53* inactivation (three-level variable) and *CKS1B* gain/amplification (three-level variable) (Fig. 3d and Supplementary Table 10). Pairwise interactions between these CN and bi-allelic covariates were tested for significance, and no significant interactions entered into the model. After adjustment for age, ISS, and study site, all previously mentioned genetic factors remained in the model, and the study site indicator was removed from the final model due to non-significance.

For both PFS and OS models, interactions between all mutational, CN, and bi-allelic inactivation factors were considered for inclusion in multivariate models both before and after adjustment for clinical effects. The final model of PFS has a cumulative *R*^2^ of 34.3% and the set of genetic factors included in the model without adjustment for age and ISS has a cumulative *R*^2^ of 18.4%. The final model of OS has a cumulative *R*^2^ of 46.5% and the set of genetic factors included in this model without adjustment for age and ISS have a cumulative *R*^2^ of 25.2%. A visualization of the contribution of individual factors in the final models of PFS and OS to the total *R*^2^ is given (Fig. 3).

### Recursive partitioning to identify high-risk Double-Hit cases

To identify patients at high risk of early progression, recursive partitioning was performed using the genetic and clinical factors identified in the final multivariate Cox model for PFS. In an initial analysis using only molecular features the first three splits of the tree identified *TP53* bi-allelic inactivation and amplification of *CKS1B* corresponding to nodes 2 and 7 (Supplementary Figure 7A). For both nodes, the log-rank *P*-value for PFS when compared to the node featuring patients with none of these features (node 6) was less than 0.05, indicating that poor PFS is associated with each of these genetic features (Supplementary Figure 7B). Based on this, *TP53* bi-allelic inactivation and amplification of *CKS1B* were considered adverse genetic features, and indicators were created for all patients for the presence of at least one or both of these factors. Of the 80 patients with at least one of the two adverse genetic factors, 77 patients had exactly one, while three had both adverse factors.

**Fig. 4:**
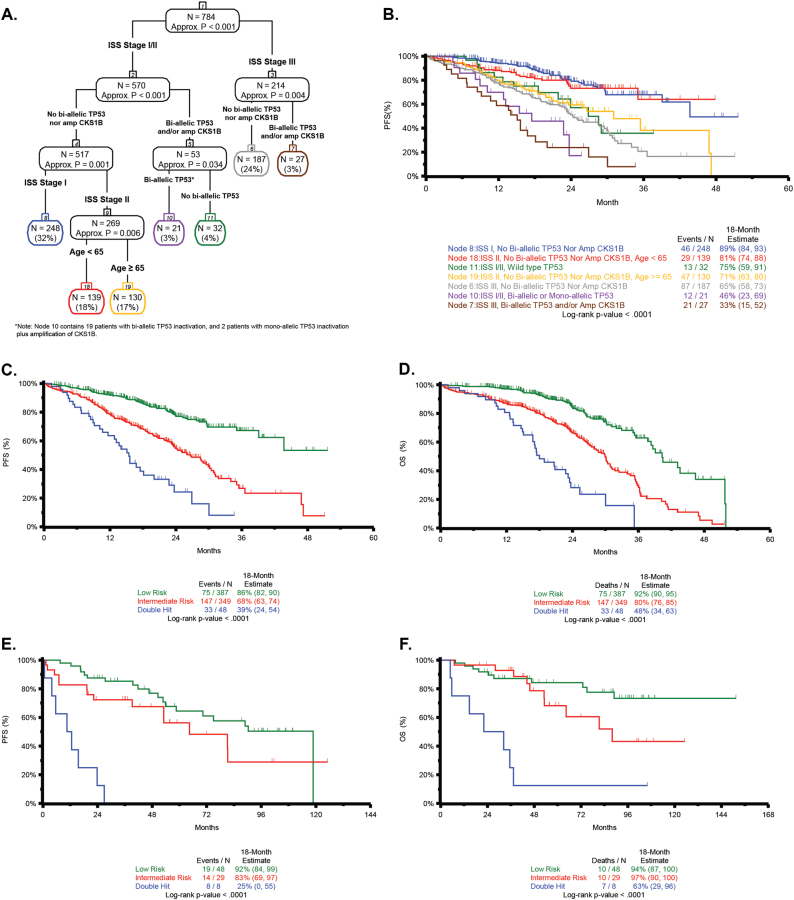
A recursive partitioning model for PFS and OS identified clinical and genomic markers associated with risk. **a** A recursive partitioning model for PFS based on the inclusion of genetic and clinical predictors, showing the terminal nodes. **b** Kaplan–Meier curves were generated for PFS for all terminal nodes of the tree. **c** Nodes with similar outcome profiles were combined to generate three risk groups. Nodes 8 and 18 were combined to designate low-risk patients (green); nodes 11, 19, and 6 were combined to designate intermediate-risk patients (red); nodes 10 and 7 were combined to designate Double-Hit patients (blue). Double-Hit comprised 6.1% of the total patient population and included patients who were either of the following: bi-allelic inactivation of *TP53* or ISS stage III with amplification of *CKS1B*. Significant differences in PFS between the risk groups are identified (*P* < 0.0001). **d** As in (**c**) with OS. **e** The risk groups identified in (**c**) were applied to a subset of Total Therapy patients (*n* = 85) with available genetic data; significantly different PFS outcomes are observed, with especially poor PFS in Double-Hit patients (*P* < 0.0001). **f** As in (**e**) with OS

Recursive partitioning was subsequently applied using both clinical features (age, ISS) and the presence of ≥1 of the two adverse genetic factors identified above to create the final tree (Fig. 4a). Nodes were assessed for PFS (Fig. 4b), and a schema for risk classification was generated by combining the resultant seven nodes into groups with similar PFS to generate three risk groups (Fig. 4c). Nodes 8 and 18 were grouped and considered low risk (49.4%), containing patients who were younger, had lower disease stage (ISS I or II), and no genetic factors. The intermediate-risk group (44.5%, nodes 11, 6, and 19) was a mixture of patients with either older age (node 19), higher disease stage without genetic factors (node 6), or who were lower stage with wild-type *TP53*, but amplification of *CKS1B* (node 11). These data are similar to the IMWG consensus on risk stratification where low-risk patients had low/intermediate disease stage and no genetic factors, and intermediate-risk was defined by either (a) ISS I plus t(4;14) or del17p, or (b) ISS III with no genetic factors [13].

Nodes 7 and 10 were grouped to generate a subset of high-risk patients referred to as Double-Hit, who had the poorest prognosis (median PFS 15.4 months, median OS 20.7 months). This group comprises 6.1% of patients and is defined by bi-allelic inactivation of *TP53* or ISS III with amplification of *CKS1B*. Significant overall differences in PFS and OS (*P* < 0.0001) were observed between these groups (Fig. 4c, d). For the Double-Hit group (*n* = 48), 27 patients (56.3%) were ISS III, 24 (50%) were ≥65 years, 30 (62.5%) had bi-allelic inactivation of *TP53*, and 21 (43.8%) had amplification of *CKS1B*. The adverse impact of these genetic features on outcomes can be appreciated when these cases are compared to patients in node 6 (ISS III, no genetic factors). The median PFS of Double-Hit patients was 9 months shorter than those in node 6 (15.4 vs. 24.4 months, respectively *P* < 0.01) and the median OS was 20.7 months vs. not reached (*P* < 0.01) (Fig. 4b).

### Validation of approach on an independent dataset

To understand how the Double-Hit subgroup performed in an independent dataset, we analyzed NDMM patients with longer follow-up derived from Total Therapy (TT) trials. A total of 85 patients (median PFS 6.25 years, median OS not reached) (Supplementary Methods, Supplementary Table 1) [22] had available clinical-sequencing and targeted-sequencing panel data, and could be segmented based on the recursive partitioning model (Fig. 4). This analysis identified a Double-Hit group of similar size and outcome, (9.4% [8/85], median PFS 11.6 months, median OS 27.2 months) (Fig. 4e, f). These results demonstrate the effectiveness of the classification of Double-Hit MM patients on an external validation set.

### Comparison to the IMWG risk classifier

**Fig. 5:**
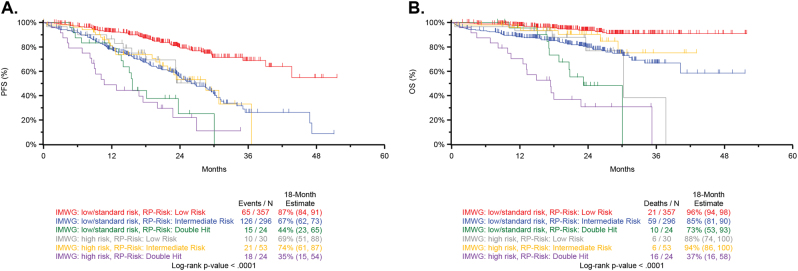
Comparison of IMWG and Double-Hit cases. **a** Patients were classified by IMWG status and recursive partitioning risk groups, as detailed in Fig. 4. Double-Hit patients have very poor PFS, whether classified as high risk by IMWG (median PFS 11-month, 18-month PFS of 35%) or low/intermediate risk by IMWG (median PFS 16-month, 18-month PFS of 44%). **b** Similar trends were observed for OS classified by both IMWG and recursive partitioning status

A comparison of Double-Hit identified by recursive partitioning to the established IMWG classifier provides an opportunity to contextualize the adverse outcome associated with Double-Hit patients [13]. The distribution of patients by IMWG risk group and recursive partitioning is provided (Supplementary Tables 1 and 11). Patients classified as Double-Hit by the recursive partitioning model had poor outcome, whether classified as high risk by IMWG criteria (*n* = 24; 18-month estimates PFS: 35%, OS: 37%) or low/standard risk (*n* = 24; PFS: 44%, OS: 73%). Additionally, patients classified as high risk by IMWG criteria, but classified as low risk (*n* = 30; PFS: 69%, OS: 88%) by recursive partitioning or intermediate risk (*n* = 53; PFS: 74%, OS: 94%) had similar outcome to patients classified as low/standard risk by IMWG, but intermediate risk by recursive partitioning (*n* = 296; PFS:67%, OS:85%) (Fig. 5).

### Molecular markers of Double-Hit MM

**Fig. 6:**
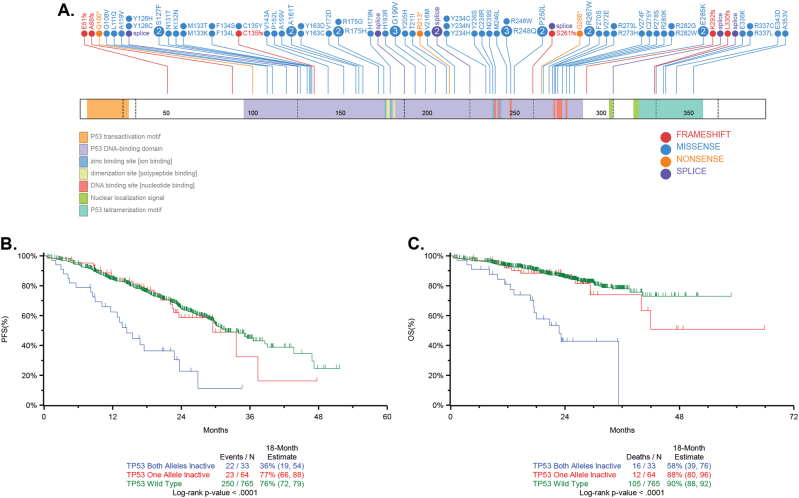
The sites of *TP53* mutation and their Impact on survival. **a** Schematic of mutations detected in *TP53*. **b** Kaplan–Meier survival curve for PFS for complete set (*n* = 863) of NDMM patients <75 years of age who had SNV and CNV results, and survival data by *TP53* bi-allelic, mono-allelic, or wild-type status. Note that this dataset is larger than the *n* = 784 dataset, since for this analysis, presence of ISS was not required. **c** OS in the same set of patients (*n* = 863)

In the full dataset, *TP53* deletion was seen in 9.0% (97/1074) and mutations in 5.5% (70/1273) of patients. Any event at *TP53* was found in 11.3% and bi-allelic events in 3.7% of patients. Mutations were predominantly found in the DNA-binding domain (80.2%, 65/81), with 7.4% (6/81) in the oligomerization domain (Fig. 6a). There were recurrent mutations in 17 codons comprising 48.1% (39/81) of mutations, with R248 (*n* = 4), R175, G199, and Y234 (all *n* = 3) being the most frequent. Missense mutations in *TP53* were seen in 77.8% (63/81), with the remaining 22.2% (18/81) being potentially protein-terminating comprising frameshift, splice site, or nonsense mutations.

Bi-allelic inactivation of *TP53* is the crucial driver of prognosis, *P* < 0.0001, when compared to wild-type or mono-allelic inactivation for both PFS and OS. Importantly, when mutations of *TP53* are taken into account, CN loss of 17p, a feature previously used to identify adverse risk [13], is not prognostically important (Fig. 6b, c). Interestingly, other studies reported similar PFS and OS in patients with bi-allelic inactivation of *TP53*, but unlike our analysis, none provided a comprehensive or compelling reason to include these features into the definition of high risk [23, 24].

**Fig. 7:**
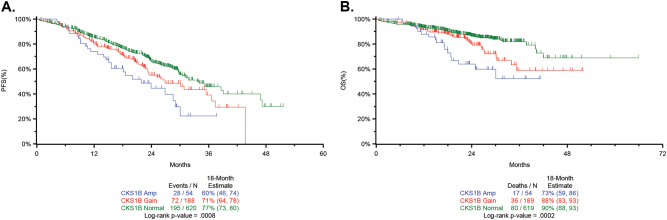
The association of gain and amplification of 1q21 with survival using *CKS1B* as the marker. **a** Kaplan–Meier survival curves for PFS based on either gain or amplification (≥4 copies) of *CKS1B* (1q21). The data are shown for the complete dataset (*n* = 863) of NDMM patients who were <75 years of age who had SNV and CNV results and survival data. Note that this dataset is larger than the *n* = 784 dataset, since for this analysis, the presence of ISS was not required. **b** OS in the same set of patients (*n* = 863)

The second genomic variable that defines the Double-Hit group is amplification (≥4 copies) of *CKS1B* in the context of ISS III. In contrast to the gain of *CKS1B* (21.9%, 189/863), the group with amplification constitutes a much smaller subset (6.3%, 54/863). In the complete dataset (*n* = 863), both gain and amplification of *CKS1B* were associated with decreased PFS and OS, but the effect was more pronounced in patients with amplification (18-month estimates, gain vs. amplification; PFS: 71 vs. 60% (*P* = 0.06; OS: 88 vs. 73%, *P* = 0.08) (Fig. 7). Other than its occurrence in a clinical group defined by ISS III, we could not identify additional adverse PFS when amp1q occurred with del17p, bi-allelic *TP53*, t(4;14), or t(14;16) (Supplementary Table 6). Interestingly, amplification of *CKS1B* did not appear to affect OS ≤ 12 months, but there was clear separation after this time, suggesting that it may have a greater effect on the intermediate group after more prolonged follow-up (Fig. 7c).

## Discussion

Using comprehensive, genome-wide analysis, we identified Double-Hit myeloma, a new, genomically defined high-risk group of patients with extremely poor outcome, despite treatment with novel therapies (18-month estimates of PFS and OS are 39% and 48%, respectively). Among 34 Double-Hit patients with available treatment data, 85% (29/34) received a ≥three-drug induction regimen with 44% (15/34) of these being a triplet (combinations of bortezomib, dexamethasone, cyclophosphamide, and lenalidomide most commonly). Compared to IMWG criteria traditionally used in the clinic, outcomes for Double-Hit patients are similar whether the patients are IMWG high risk (18-month estimates of PFS and OS are 35% and 37%, respectively) or IMWG low/standard risk (18-month estimates of PFS and OS are 44% and 73%, respectively). The similarly poor outcomes of these two groups, especially when compared to other possible classifications by IMWG risk and recursive partitioning, suggests that existing classifiers of high risk fail to identify some of the patients at greatest risk for poor clinical outcome and that defining Double-Hit constitutes a significant step forward. Given the frequency of gain and amplification of 1q (21.9% and 6.1%, respectively in this study) and the impact on the outcome, either addition of these features, or revision to classify them as part of the high-risk definition in the R-ISS and IMWG risk stratification methods could improve the current risk criteria [13, 14]. It seems that making the distinction between gain and amp1q is the key to identifying a high-risk group of patients, and that in future studies, a clear distinction between the two states should be discerned.

Importantly, the number of prognostic molecular features required to identify the Double-Hit group is small, making their inclusion into clinically valuable risk stratification approaches relatively simple. There are only two highly penetrant genetic features which define the Double-Hit status. However, the molecular criteria rely on discerning subtle differences in *TP53* (bi-allelic vs. deletion) and 1q (amp vs. gain), so it is important that diagnostic tests can easily discriminate between them. The ease and speed of detection of DNA markers are key features that are relevant in the clinic, and the limited number of variables required for identification of Double-Hit patients is an advantage. It is important to not just determine the CN status of 17p, but also determine the mutational status of *TP53*. In addition, fluorescence in situ hybridization (FISH) probes may not be sensitive enough to identify small deletions in *TP53*, such as exonic or promoter deletions, resulting in misclassification of patients. As such, modern molecular tests should be used, such as sequencing panels to accurately determine *TP53* status. These need not be myeloma-specific panels, as many vendors provide kits or services for *TP53* [25, 26]. NGS-based assays can detect *TP53* mutation/deletion and gain/amplification of 1q when present in >30% of cells, allowing efficient detection of prognostically important variants at levels comparable to interphase FISH. NGS-based definition of these abnormalities will also generate a more homogenously defined population by removing variability generated by cytogenetic frequency calls, allowing more reproducibility.

Using the largest dataset available, we show that bi-allelic alteration of *TP53* is present in 3.7% of NDMM and is associated with very poor outcome. Bi-allelic inactivation of *TP53* has been reported higher at relapse, having been reported at 21–26% [27–29], and is also observed in other hematological malignancies, including chronic lymphoctic leukemia (CLL) and acute lymphoblastic leukemia (ALL) [30, 31]. In all, *TP53* mutations are reported at 15.7% and, like myeloma, patients with bi-allelic inactivation have shorter OS compared to either mutation or deletion alone [31].

In NDMM, we show that deletion of 17p alone is not prognostic; in fact when mutation in *TP53* is accounted for, monosomy 17p alone has no prognostic value. The prognostic relevance of this highlights the need for sequencing of *TP53* in diagnostic laboratories. Previous studies detected *TP53* mutations in 3–8% of myeloma patients, varying by dataset size and technique used [15, 16, 32]. We show that mutation of *TP53* is present in 5.5% of patients. Similar to other cancers [33, 34], mutations in *TP53* are predominantly missense mutations in the DNA-binding domain. Nonsense or frameshift mutations were relatively rare, given that *TP53* is a tumor suppressor gene, and the predominance of missense mutations may indicate altered function of *TP53* rather than complete inactivation.

CN gains of 1q have long been associated with poor outcome, and it is known that as the number of copies of 1q increases, there is an association with worse outcome [35, 36]. Definitions of amp1q vary throughout the literature, making it difficult to determine the true prognostic impact of gain vs. amplification. We used ≥4 copies to define amp1q and show an association with significantly poorer outcome, especially on a background of ISS III. The biological meaning of amp1q is difficult to determine; some studies have suggested overexpression of specific particular genes that are overexpressed on 1q, or mechanisms of genome instability that cause the amplification through translocations and hypomethlation [5, 36–39].

The possibility remains that additional prognostically important genomic features contributing to early relapse could be identified in future; however, we believe that this is unlikely, given the size of this dataset. Also, this analysis focused on DNA-based features; however, an integrated analysis that includes whole-transcriptome data is ongoing. Despite its size, the study currently lacks the power to exclude subtle contributions of genetic variables to long-term survival because of limited follow-up; however, this was not the purpose of the analysis. Instead, we investigated whether patients with extremely poor outcome at presentation could be identified using molecular features where experimental therapeutic strategies could be explored.

An important future direction will be discussion and engagement with myeloma working groups and integration of these data into consensus risk stratification criteria to ensure communication and adoption throughout the global myeloma community. In addition, engagement with regulatory agencies will be needed to explore inclusion of the Double-Hit group in clinical studies. These patients can be readily identified using NGS-based assays, providing an opportunity to evaluate innovative therapeutic strategies, such as chimeric antigen receptor T cells, to address their unmet medical need.

## Electronic supplementary material

Supplementary Material
