# Supplementary material for: A high-risk, Double-Hit, group of newly diagnosed myeloma identified by genomic analysis
Source: Leukemia. 2018 Jul 2;33(1):159–70. doi: 10.1038/s41375-018-0196-8 (PMC6326953; doi:10.1038/s41375-018-0196-8)
Supplement: Supplementary file 1 — Supplementary Material [file 41375_2018_196_MOESM1_ESM.pdf]

A High-Risk, Double Hit Group of Newly Diagnosed Myeloma Identified by Genomic Analysis

Supplementary Document

Brian A. Walker[1]*, Konstantinos Mavrommatis[2]*, Christopher P. Wardell[1]*, T. Cody Ashby[1], Michael Bauer[1], Faith Davies[1], Adam Rosenthal[3], Hongwei Wang[3], Pingping Qu[3], Antje Hoering[3], Mehmet Samur[4], Fadi Towfic[5], Maria Ortiz[6], Erin Flynt[5], Zhinuan Yu[5], Zhihong Yang[5], Dan Rozelle[7], John Obenauer[7], Matthew Trotter[6], Daniel Auclair[8], Jonathan Keats[9], Niccolo Bolli[10], Mariateresa Fulciniti[4], Raphael Szalat[4], Phillipe Moreau[11], Brian Durie[12], A. Keith Stewart[13] Hartmut Goldschmidt[14], Marc S. Raab[14,15], Hermann Einsele[16], Pieter Sonneveld[17], Jesus San Miguel[18], Sagar Lonial[19], Graham H. Jackson[20], Kenneth C. Anderson[4], Herve Avet-Loiseau[21,22], Nikhil Munshi[4]**, Anjan Thakurta[5]**, Gareth Morgan[1]**.

*These authors contributed equally to this work and are co-lead authors.
**These authors contributed equally to this work and are co-senior authors.

1 Myeloma Institute, University of Arkansas for Medical Sciences, Little Rock, AR, USA.
2 Celgene Corporation, San Francisco, CA, USA.
3 Cancer Research and Biostatistics, Seattle, WA, USA.
4 Dana-Farber Cancer Institute, Harvard Medical School, Boston, MA, USA.
5 Celgene Corporation, Summit, NJ, USA.
6 Celgene Institute of Translational Research Europe, Sevilla, Spain.
7 Rancho BioSciences, San Diego, CA, USA.
8 Multiple Myeloma Research Foundation, Norwalk, CT, USA.
9 Translational Genomics Research Institute, Phoenix, AZ, USA.
10 University of Milan, Italy.
11 University of Nantes, France.
12 Cedars-Sinai Samuel Oschin Cancer Center, Los Angeles, CA, USA.
13 Department of Hematology, Mayo Clinic, Scottsdale, AZ, USA.
14 Department of Medicine V, Hematology and Oncology, University Hospital of Heidelberg, Heidelberg, Germany.
15 German Cancer Research Center (DKFZ), Heidelberg, Heidelberg, Germany.
16 Department of Internal Medicine II, Wurzburg University, Wurzburg, Germany.
17 Department of Hematology, Erasmus MC Cancer Institute, Rotterdam, The Netherlands.
18 Clinica Universidad de Navarra, Centro Investigacion Medica Aplicada (CIMA), IDISNA, CIBERONC, Pamplona, Spain.
19 Winship Cancer Institute , Emory University, Atlanta, GA , USA.
20 Department of Haematology, Newcastle University, Newcastle, UK.
21 Centre de Recherche en Cancérologie de Toulouse Institut National de la Santé et de la Recherche Médicale U1037, Toulouse, France.
22 L'Institut Universitaire du Cancer de Toulouse Oncopole, Centre Hospitalier Universitaire, Toulouse, France.

Table of Contents

Supplementary Clinical Outcomes 4
Patients and datasets 4
Genomic Methods 4
Patient and molecular subtypes, Datasets, and Variant Calling 5
Statistical Methods 5
General methodology 5
Development of APOBEC signature, LOH percentage, homologous recombination deficiency, and copy number cluster 5
Covariates used for modeling outcome 7
Covariate selection among highly concordant covariates 7
Cox regression for progression free survival and overall survival 8
Recursive partitioning to determine risk groups 8
Supplementary References 10
Supplementary Figures 11
Supplementary Figure 1. Distribution of clinical data 11
Supplementary Figure 2. Survival curves dependent upon ISS stage, age and IMWG risk status. 12
Supplementary Figure 3. Datasets available for analyses. 13
Supplementary Figure 4. The impact of cytogenetic subgroups on survival. 14
Supplementary Figure 5. The association of deletion of 1p markers (*CDKN2C*, *RPL5* and *FAM46C*) with PFS (A-C) and OS (D-F), the association of deletion of any 1p marker (*CDKN2C, RPL5, FAM46C*) on clinical outcome PFS (G) and OS (H) (n=863)) 15
Supplementary Figure 6. The interaction of deletion of 1p markers (*CDKN2C/RPL5/FAM46C*) and gain/amp of 1q with PFS (A-C) and OS (D-F). The interaction of any 1p deletion in *CDKN2C/RPL5/FAM46C* and 1q gain/amplification on PFS (G) and OS (H) (n=863). 16
Supplementary Figure 7. Recursive partitioning analysis for PFS. 17
Supplementary Tables 18
Supplementary Table 1. Number of samples and their baseline characteristics 18
Supplementary Table 2. Frequency and association of survival with bi-allelic inactivation of tumor suppressor genes 19
Supplementary Table 3. Significantly mutated genes (according to MutSigCV) and their association with survival. 20
Supplementary Table 4. Recurrent copy number abnormalities and their association with survival. 21
Supplementary Table 5. The association of chromosomal translocations and copy number clusters with survival. 22
Supplementary Table 6. The association of selected interactions with survival by univariate Cox analysis. 23
Supplementary Table 7. Univariate PFS associations and hazard ratios. 24
Supplementary Table 8. Univariate OS associations and hazard ratios. 25

Supplementary Table 9. Multivariate PFS associations and hazard ratios. 26
Supplementary Table 10. Multivariate OS associations and hazard ratios. 27
Supplementary Table 11. Comparison of patients being low, intermediate, Double Hit Risk in the recursive partitioning model by either ISS or IMWG in the MGP patient set. 28
Supplementary Table 12. Indicators used for chromosomes featuring multiple genes with extremely high concordance in copy number data. 29

Supplementary Clinical Outcomes

Patients and datasets

This work is based on an analysis of a set of NDMM cases with clinical and outcome data associated with whole exome sequencing (WES; n=1,273). The data were derived from the Myeloma XI trial, Dana-Faber Cancer Institute (DFCI)/Intergroupe Francophone du Myelome (IFM), and the Multiple Myeloma Research Foundation (MMRF) CoMMpass study, all of which have been reported elsewhere, **Supplementary Figure 1** and **Table 1**.[1-7] The Myeloma XI study cases included patients treated with either a triplet immunomodulatory drugs (IMiDs® agent) based induction with or without high dose treatment and included patients randomized to lenalidomide maintenance. The DFCI/IFM 2009 study included patients younger than 65 years of age who received three cycles of lenalidomide, bortezomib plus dexamethasone induction therapy, followed by stem cell mobilization and either a) five additional cycles of triplet therapy plus maintenance, or b) high dose therapy followed by 2 cycles of triplet therapy and maintenance.[5] CoMMpass is a prospective, longitudinal, observational, investigator's choice regimen study of NDMM that included patients from the United States, Canada, Italy, and Spain. First line regimens in CoMMpass included singlets (5%; bortezomib [3%] or lenalidomide [2%]), doublets (33%; bortezomib+dexamethasone [dex] [19%], lenalidomide+dex [9%], or carfilzomib+dex [3%]), or triplets (61%; bortezomib+lenalidomide+dex [32%], bortezomib+cyclophosphamide+dex [20%], bortezomib+melphalan+prednisone [4%], or carfilzomib+lenalidomide+dex [2%]) https://www.themmrf.org/research-partners/mmrf-data-bank/the-mmrf-commpass-study/. For CoMMpass, genomic data in these analyses were derived from the IA9 data cut, while clinical follow-up and outcomes were derived from IA10.

Patients age ≥75 were associated with poor outcome irrespective of genetic background and were, therefore, excluded from survival analyses. A set of 784 patients with complete clinical and molecular data was available for survival analysis, and was used to develop and validate a classification schema based on recursive partitioning analyses. The median follow-up of the analysis dataset was 22.9 months, range 0 - 52 months, with a median progression free survival (PFS) of 31.2 months (median overall [OS] has not yet been reached). Of the 784 patients with available data, 675 either completed at least one year of therapy or experienced a PFS event (progression/relapse or death) within the first year; of these 675 patients, 116 (17.2%) progressed or died within the first year. The survival data patterns for OS and PFS were generally representative of well-established clinical and cytogenetic subgroups of NDMM including age, ISS, and International Myeloma Working Group (IMWG) risk groups, **Supplementary Figure 2**.

Genomic Methods

All code associated with the analyses included herein is provided on GitHub under https://github.com/celgene-research/mgp_doublehit. Our genomic pipeline code is provided under https://github.com/celgene-research/mgp_ngs.

Patient and molecular subtypes, Datasets, and Variant Calling

The datasets analyzed in this analysis are summarized, **Supplementary Figure 3**. The samples were uniformly classified at a molecular level using the genetic material, all methods are described in detail in Walker et al "Identification of Novel Mutational Drivers Reveals Oncogene Dependencies in Multiple Myeloma"

Sequencing data have been deposited in the European Genomic Archive under the accession numbers EGAS00001001147, EGAS00001000036, EGAS00001002859 or at dbGAP under Accession phs000748.v5.p4.

Statistical Methods

General methodology

The Kaplan-Meier method was used to estimate time-to-event distributions. A Cox proportional hazards regression procedure was used to select models and estimate the effects of important covariates in time-to-event outcome models. Cumulative R^2 was calculated[8] for factors which entered regression models based on the order in which they entered the models. Comparisons of distributions and key summary statistical measures for covariates were performed using Fisher's exact test.

Multivariate Cox models for PFS and OS were generated via stepwise regression using genetic factors with, the models subsequently being adjusted for age, ISS, and study site. The final Cox model consisted only of statistically significant factors ($P \leq 0.05$) after adjustment for the inclusion of both genetic and clinical factors. All mutation and copy number factors were investigated for high concordance, and all possible pairs of interactions for mutation and copy number factors selected in the final multivariate Cox models were tested for significance.

Development of APOBEC signature, LOH percentage, homologous recombination deficiency, and copy number cluster

A detailed description of methods and results can be found elsewhere.[9] Mutational signatures were called using non-negative matrix factorization (NMF) with counts per sample calculated for the six possible SNV types and the 16 possible 2-base sequence contexts, creating a table with 1,273 rows and 96 columns. The R package "NMF" was used for all calculations.[10] The number of signatures was determined by running 50 iterations of the algorithm for 2-10 signatures. A number of signatures was chosen that maximized the cophenetic distance and dispersion values. One thousand (1,000) iterations of the algorithm were run for that number of signatures. Cosine similarity was used to determine the Sanger signatures that were closest to the detected signatures.[11]

The extent of LOH was defined using copy number data by the following process:

i) Categorize the copy number of each gene based on calls produced by Control-FREEC (0=homozygous deletion; 1=deletion; 2=normal; 3=gain; 4=amplification).

ii) Consider each arm of each chromosome separately. For metacentric and submetacentric chromosomes consider both the *p* and *q* portions, but only one arm in the case of acrocentric chromosomes.

iii) Segment each chromosomal arm based on the run-length encoding of the copy number status of genes. This step is similar to the construction of the CIGAR strings found in the Sequence Alignment/Map (SAM) format. For example, if the portion of interest has 10 normal genes, 100 deletions in genes and 10 gains in genes, the string will be 10N100D10G. Each of these are considered a copy number region.

iv) If a single copy number region contains the deletion of more than half of the genes (50%) on a chromosomal arm, the entire chromosomal arm is disqualified from analysis and counted as a large-scale deletion.

v) In addition, chromosomal arms with fewer than 3 copy number regions are disqualified from analysis.

vi) The copy number region that is closest to the telomere is ignored (i.e., the first region if it is located on the *p* arm of the chromosome or last region if it is located on the *q* arm of the chromosome).

vii) The extent of LOH for a region (ranging from a chromosomal arm to the entire genome) is defined by dividing the number of genes that are deleted by the total number of genes.

For example, given the following string 10D10G10D10A10N10D for the *p* arm of a chromosome, the first copy number region would be ignored (step vi), leaving 50 genes in total and 20 genes that are deleted (step vii). This results in 40% (20/50) LOH for that chromosomal arm.

The results generated were then used to define the optimum cut point of 4.6%. To produce this cut point, the data were dichotomized into 2 groups based on the LOH percentage for a range of cut points and survival analysis was performed. The optimal cut point was defined as that which gave the maximum logrank test statistic among all those computed. This was performed for both OS and PFS, which gave very similar results. Seventy-seven (77) samples (7.0%) were above this cut point. The distribution of an excess of LOH using this cut point was examined across the major molecular and clinical subgroups of myeloma. Comparisons were performed using Wilcox tests and the false discovery rate method of multiple testing correction was applied.

Homologous recombination deficiency mutations have been used to generate a signature that correlates with sensitivity to PARP inhibitors in ovarian cancer.[12] This signature comprises 15 genes involved in DNA repair pathways (*ATM, BARD1, BRCA1, BRCA2, BRIP1, CDK12, CHEK2, FANCA, NBN, PALB2, RAD51, RAD51B, RAD51C, RAD51D, RAD54L*). Mutations in any of these genes were used to define patients that may have deficiency in homologous recombination.

The 39 copy number features were merged on the odd numbered chromosome whenever correlation was > 90% (by selecting a marker on the *q* arm) to avoid redundant information biasing the clustering result. This resulted in 29 features. Data were scaled using the scale function in R. To avoid the HRD features (i.e., gains on chromosomes 3, 5, 7, 9, 15, and 19) in samples overshadowing features in other samples, the data was split into two sets, hyperdiploid (HRD) and non-hyperdiploid

(nHRD), via kmeans (k = 2). To determine the optimal number of clusters in each set the sigclust package in R was used. The k-means algorithm was iteratively run on each set, starting with $k = 2$ and incrementing by one each interaction. During the iteration, to ensure that the result was significant, sigclust was run pairwise on all clusters produced by the algorithm. The iteration stopped at the largest k where all pairs of clusters were significantly different. For the HRD set this was at k = 2 and for the nHRD set this was at k = 7.

Covariates used for modeling outcome

Variables for potential selection in Cox regression analyses included age, ISS stage, study site, IGH translocations, hyperdiploidy, MYC translocation, APOBEC signature, loss of heterozygosity (LOH) percentage, homologous recombination deficiency, copy number cluster, mutational data, copy number data, and bi-allelic inactivation data. Cut-points were used to create dichotomous variables for age and LOH. For age, survival was compared in 5-year intervals, and a cut-point of 65 years was selected. For LOH%, an optimal cut-point for the creation of a binary indicator was determined using the running log-rank test based on PFS.[7]

Several covariates had more than two factor levels for which comparisons were desired; these included ISS stage (three stages), CN-cluster (nine different clusters), some copy number data (normal, gain, and amplification), and bi-allelic inactivation data (wild type, one allele inactivated, and both alleles inactivated). Each ISS stage and each CN-cluster was considered a different factor level for analyses; the reference levels chosen for comparisons within these variables were ISS Stage I and CN-cluster 2, respectively. For bi-allelic inactivation data, levels considered for comparison were wild type, one-allele inactivation, and bi-allelic inactivation; wild type was considered the reference level for analysis. For copy number data for which loss was considered, dichotomous variables comparing loss to all other cases were created. For copy number data where gain and/or amplification were considered, gain and amplification were collectively compared to all other cases on the typically gained chromosomes in high risk disease (HRD). For 1q (*CKS1B*), the effect of both gain and amplification were compared separately against all other cases using a three-level factor.

Covariate selection among highly concordant covariates

Several cases within the data featured at least two of mutational data, copy number data, and bi-allelic data at the same gene; these features were typically highly concordant. In cases with multiple genetic data types for the same gene, copy number data was used when a gene had available copy number data; bi-allelic inactivation data was used when a gene had available bi-allelic inactivation data but not available copy number data; and mutational data was used otherwise. An exception was *TP53*, for which bi-allelic data was used rather than copy number data. Additionally, several chromosome arms featured more than one gene with copy number data available; in these cases, the dichotomous gain or loss variables typically had extremely high concordance for these genes, **Supplementary Table 12**. Indicators were created to

indicate the presence of gain or loss of any of the genes on these chromosome arms, and these indicators were used instead of individual genes on those chromosome arms for all multivariate analyses.

Cox regression for progression free survival and overall survival

All Cox regression analyses were performed on the complete dataset (n=784). Univariate Cox regression was first performed on all available covariates, and on selected interactions as determined by researchers. Multivariate Cox regression was then employed using stepwise selection such that a covariate must have been significant at the 0.1 level to enter the model, and must have been significant at the 0.05 level to remain in the model.

As a major goal of this analysis was to identify the molecular factors associated with outcome, the general approach to multivariate Cox modeling was to adjust a model obtained by featuring only molecular features for age, ISS stage, and study site to obtain a final composite model for outcome. All possible pairs of interactions among genetic factors selected by stepwise Cox regression were tested for significance and included if significant. Any factors (both genetic and clinical) found as significant (Wald $P \leq 0.05$) in the model but no longer significant after adjustment for age, ISS stage and study site were removed from the final composite model such that the final models presented consist of only significant factors after adjustment for age (<65 vs ≥65 years), ISS stage, and study site. Only one factor per gene or chromosome arm was considered for modeling as previously described, and additionally, all factors in both the model featuring only molecular features and the final composite model were checked for especially high concordance.

Additionally, cumulative R^2, the percentage of total model variance explained by a factor or set of factors, was computed for each model using the methodology of O'Quigley et al.[8] for the calculation of R^2 in the presence of censored data. Results are presented for each regression model based on the order in which variables were entered into the regression models. For full regression models adjusted for clinical factors (ISS stage and age), this allows for an assessment of the variance explained by only genomic factors, as well as the variance explained upon the addition of clinical factors to these genomic-factor models.

Recursive partitioning to determine risk groups

To develop a classifier of patient risk based on both genetic and clinical factors, recursive partitioning was applied using the **RLSplit** package in R. A recursive partitioning analysis was performed on the dataset using all available genetic factors to classify patients such that especially adverse genetic factors could be identified to find patients with similarly poor outcomes based on Kaplan-Meier analysis and log-rank tests between the nodes found through the analysis. Once these adverse genetic factors were identified, a subsequent recursive partitioning analysis was performed using the presence of one or more of these especially adverse genetic factors, as well as age and ISS stage, to identify potential risk groups for patients based on Kaplan-

Meier analysis and log-rank tests between the nodes of this analysis. Parameters used in the variable selection included a minimum node size of 20, a maximum censored percentage of 80%, and a penalty of 4 (equivalent to preserving splits with approximate local p-value of 0.05 or less).

Supplementary References

1. Walker BA, Boyle EM, Wardell CP, et al. Mutational Spectrum, Copy Number Changes, and Outcome: Results of a Sequencing Study of Patients With Newly Diagnosed Myeloma. J Clin Oncol 2015;33:3911-20.
2. Bolli N, Avet-Loiseau H, Wedge DC, et al. Heterogeneity of genomic evolution and mutational profiles in multiple myeloma. Nat Commun 2014;5:2997.
3. Lagana A, Perumal D, Melnekoff D, et al. Integrative network analysis identifies novel drivers of pathogenesis and progression in newly diagnosed multiple myeloma. Leukemia 2017.
4. Keats J, Speyer G, Christofferson A, et al. Interim Analysis of the MMRF CoMMpass Study: Comprehensive Characterization of Multiple Myeloma Patients at Diagnosis Reveals Distinct Molecular Subtypes and Clinical Outcomes. Clinical Lymphoma, Myeloma and Leukemia 2015;15:e44-e5.
5. Attal M, Lauwers-Cances V, Hulin C, et al. Lenalidomide, Bortezomib, and Dexamethasone with Transplantation for Myeloma. N Engl J Med 2017;376:1311-20.
6. Jackson G, Davies FE, Pawlyn C, et al. Lenalidomide Maintenance Significantly Improves Outcomes Compared to Observation Irrespective of Cytogenetic Risk: Results of the Myeloma XI Trial. Blood 2017;130:436-.
7. Pawlyn C, Davies FE, Cairns D, et al. Continuous Treatment with Lenalidomide Improves Outcomes in Newly Diagnosed Myeloma Patients Not Eligible for Autologous Stem Cell Transplant: Results of the Myeloma XI Trial. Blood 2017;130:1854-.
8. O'Quigley J, Xu R, Stare J. Explained randomness in proportional hazards models. Stat Med 2005;24:479-89.
9. Walker BA, Mavrommatis K, Wardell CP, et al. Identification of novel mutational drivers reveals oncogene dependencies in multiple myeloma. Blood 2018;Submitted for publication.
10. Gaujoux R, Seoighe C. A flexible R package for nonnegative matrix factorization. BMC Bioinformatics 2010;11:367.
11. Alexandrov LB, Nik-Zainal S, Wedge DC, et al. Signatures of mutational processes in human cancer. Nature 2013;500:415.
12. Swisher EM, Lin KK, Oza AM, et al. Rucaparib in relapsed, platinum-sensitive high-grade ovarian carcinoma (ARIEL2 Part 1): an international, multicentre, open-label, phase 2 trial. The Lancet Oncology 2017;18:75-87.

Supplementary Figures

A.

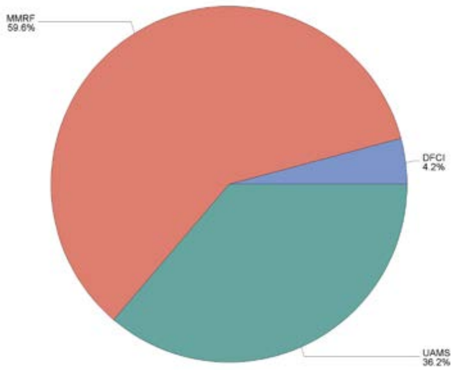

B.

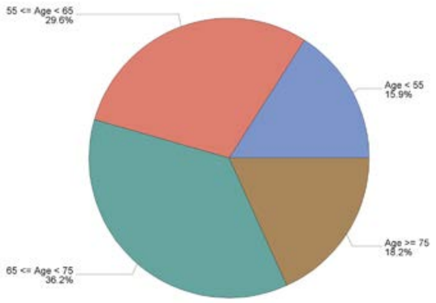

C.

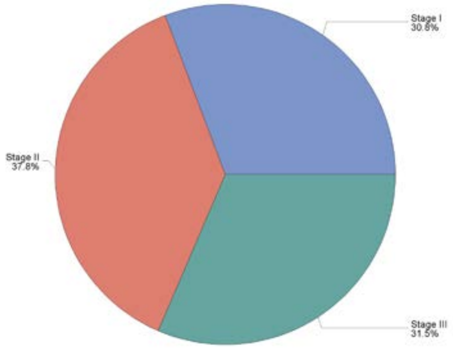

D.

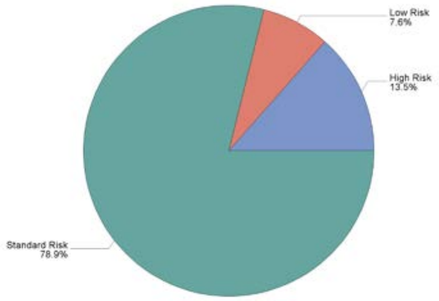

Supplementary Figure 1. Distribution of clinical data

A. The percentage of the samples contributed by each center (n=1273). B. The age distribution of patients (n=1273). C. The percentage of patients in each ISS group (n=1170). D. The percentage of patients in each IMWG risk group (n=1089).

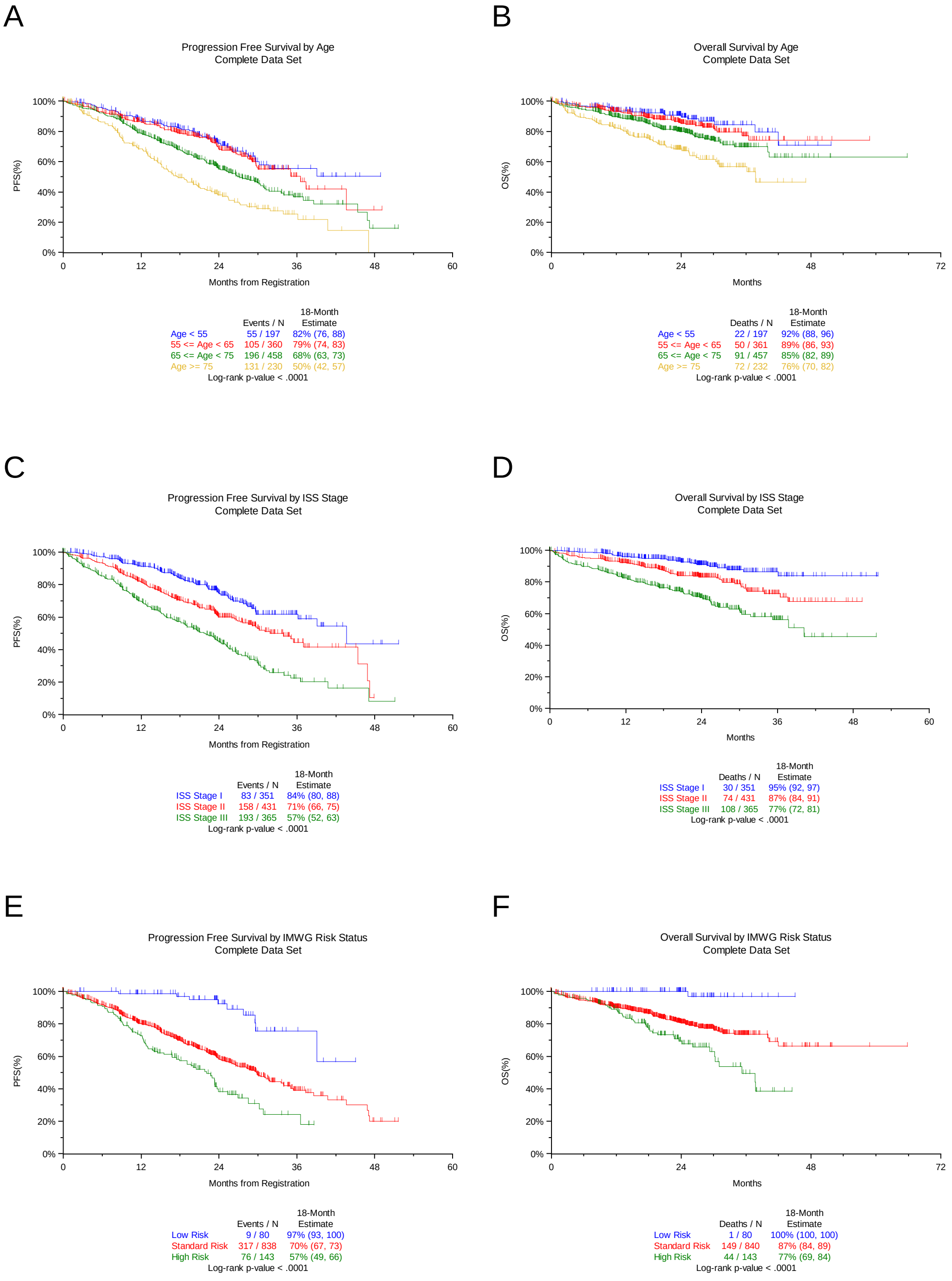

Supplementary Figure 2. Survival curves dependent upon ISS stage, age and IMWG risk status.

Kaplan-Meier curves for clinical subgroups, including age (A, PFS n=1245; B, OS n=1247), ISS stage (C, PFS n=1147; D, OS n=1147), and IMWG risk status (E, PFS n=1061; F, OS n=1063) in patients with whole exome sequencing data. All survival data was censored at four years from enrollment to account for major differences in duration of follow-up data between studies.

A.

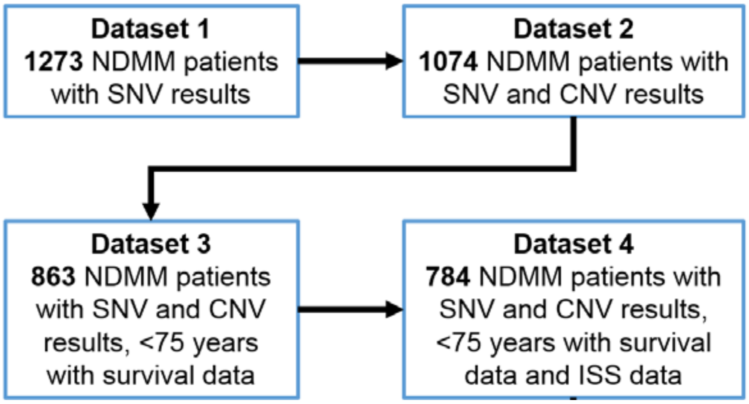

B.

Data type	Whole Data Set	Data Subset 2	Data Subset 3	Data subset 4
**Newly diagnosed MM**	1273	1074	863	784
**PFS/OS survival data**	1245	1046	863	784
**ISS**	1195	977	785	784
**Under age 75**	1075	915	863	784
**CNV passing QC**	1074	1074	863	784
**SNV/indel calls**	1273	1074	863	784
**Translocation calls**	1268	1070	863	784
**MMRF patients**	759	696	569	553
**MYXI patients**	461	326	248	231
**DFCI patients**	53	52	46	0

Supplementary Figure 3. Datasets available for analyses.
The whole data set was composed of 1273 samples from newly diagnosed MM patients with exome sequencing data. From these data, three subsets were taken, the first (n=1074) was based on copy number data that passed quality control, and the second (n=863) was based on patients under 75 who had ISS and the third (n=784) was based on also having ISS data.

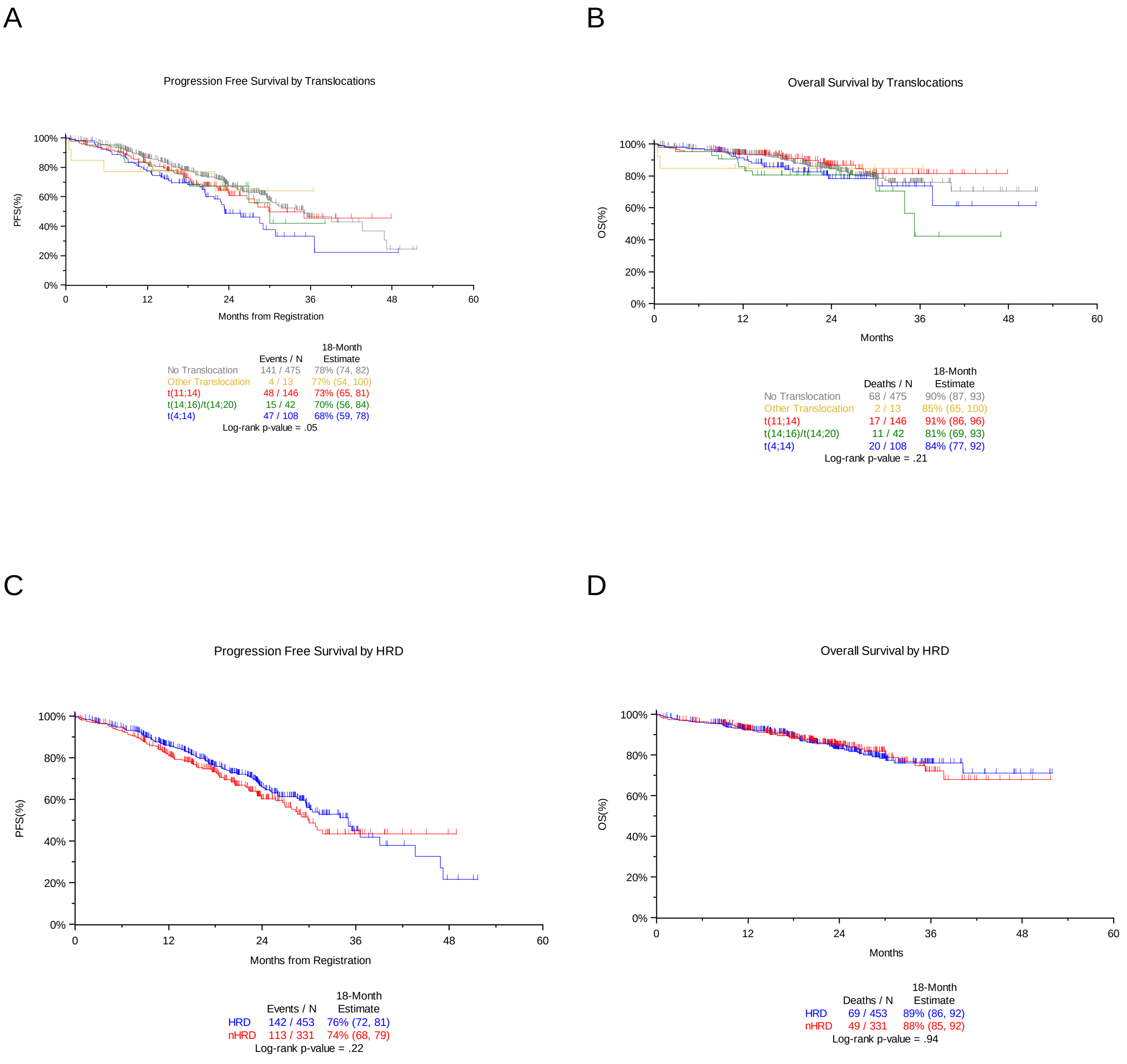

Supplementary Figure 4. The impact of cytogenetic subgroups on survival.
Kaplan-Meier survival curves for PFS and OS for translocation subgroups (A and B) and hyperdiploidy (C and D); dataset n=784 patients with ISS, survival and copy number). Samples with t(14;16) or t(14;20) were combined.

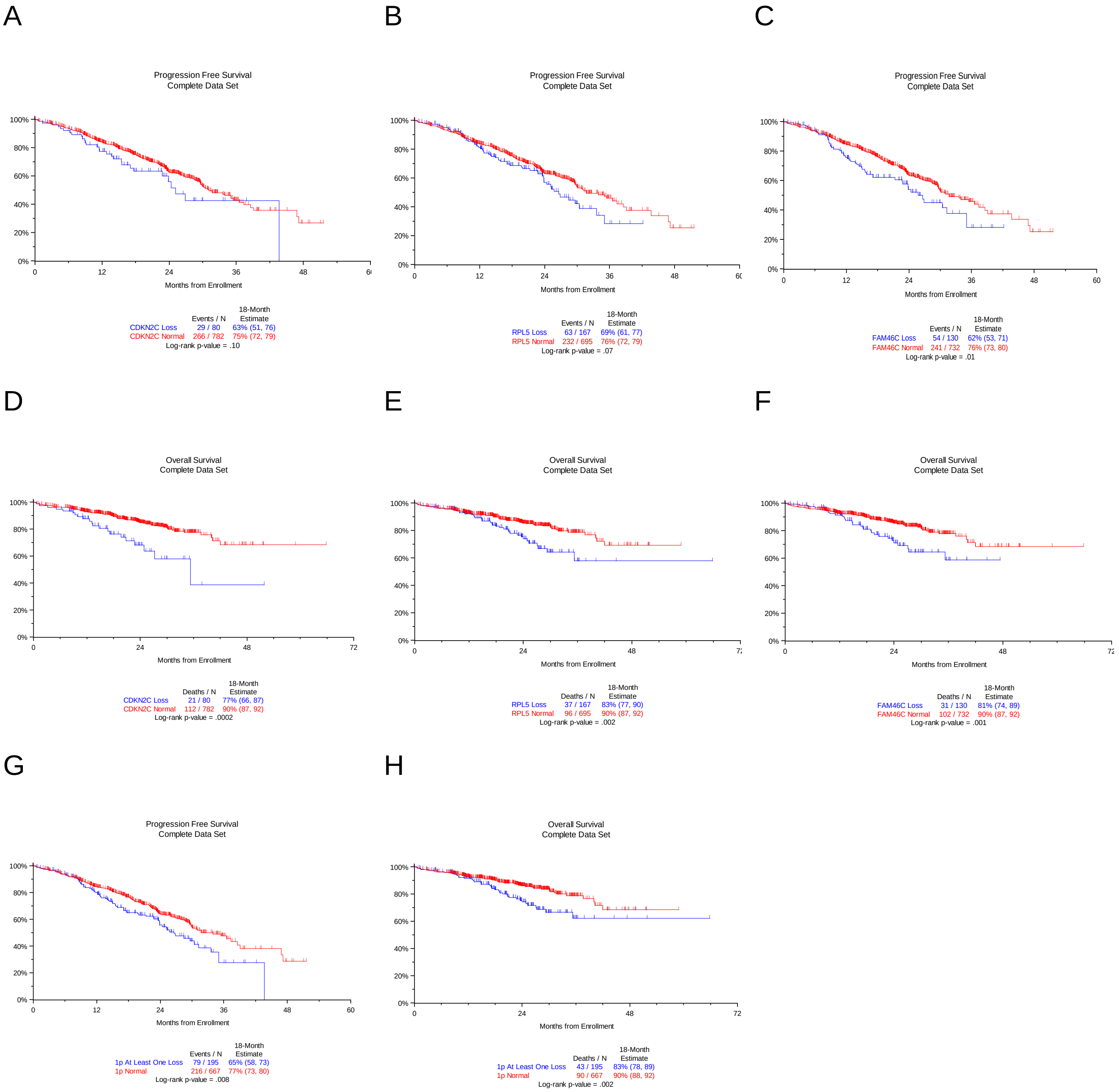

Supplementary Figure 5. The association of deletion of 1p markers (*CDKN2C*, *RPL5* and *FAM46C*) with PFS (A-C) and OS (D-F), the association of deletion of any 1p marker (*CDKN2C, RPL5, FAM46C*) on clinical outcome PFS (G) and OS (H) (n=863))

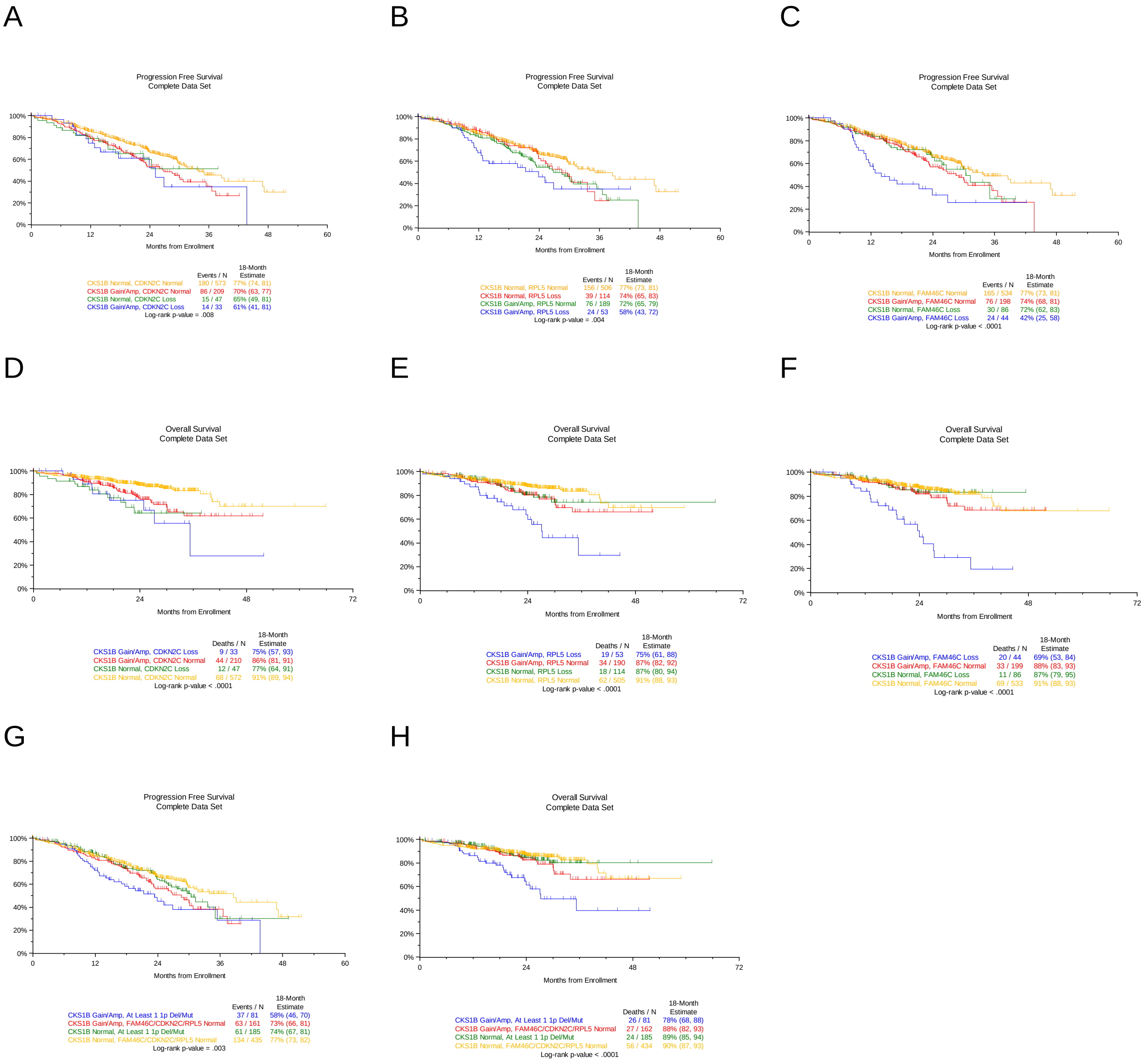

Supplementary Figure 6. The interaction of deletion of 1p markers (*CDKN2C/RPL5/FAM46C*) and gain/amp of 1q with PFS (A-C) and OS (D-F). The interaction of any 1p deletion in *CDKN2C/RPL5/FAM46C* and 1q gain/amplification on PFS (G) and OS (H) (n=863).

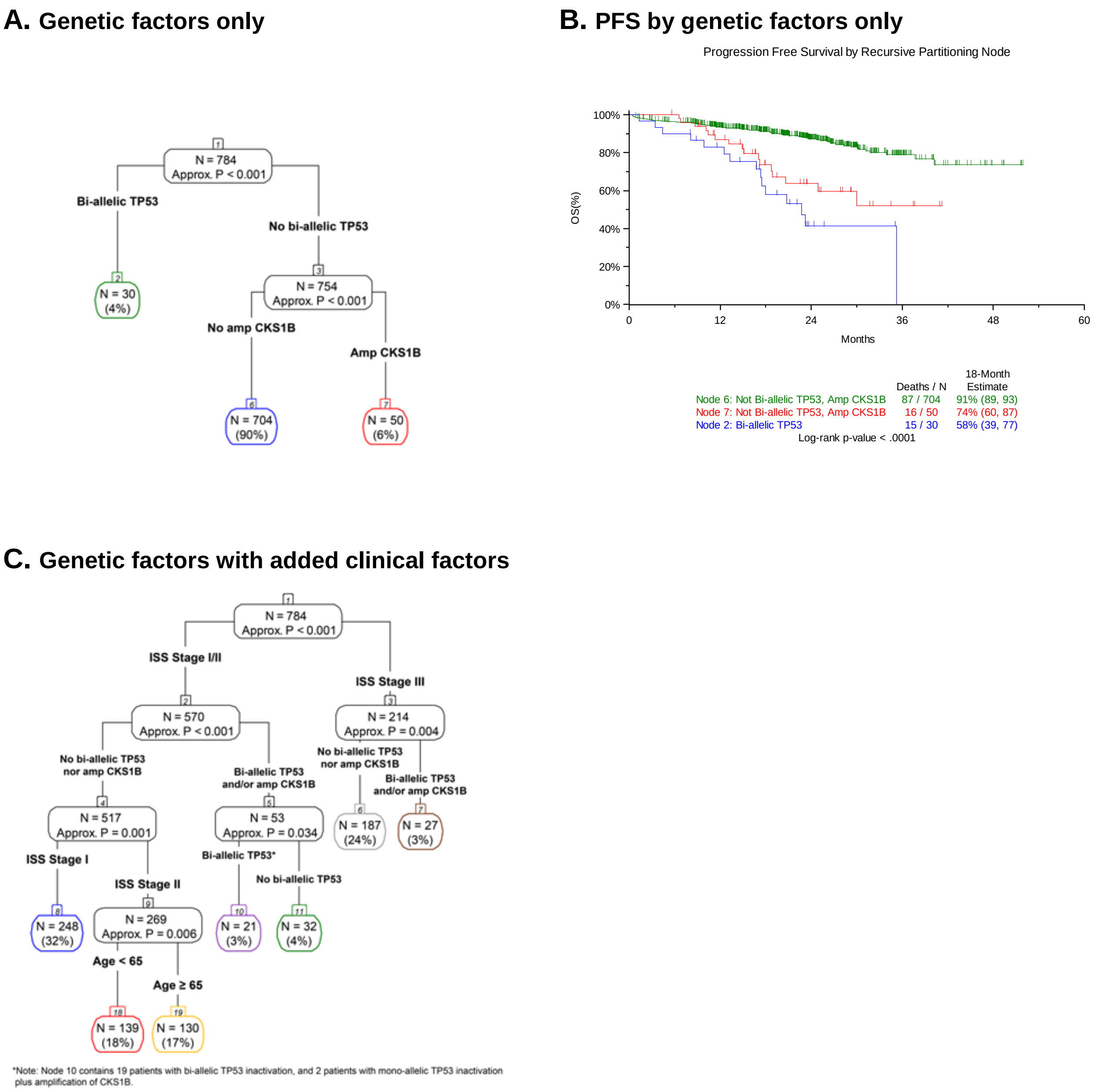

Supplementary Figure 7. Recursive partitioning analysis for PFS.

A. Recursive partitioning analysis for genetic risk factors. All factors considered for multivariate Cox modeling for outcome were used as candidates for partitioning in this model. **B.** PFS for genetic factors identified in the nodes in A; it was observed that *TP53* bi-allelic inactivation, amplification of *CKS1B*, and t(4;14) had especially poor outcome, and these three factors were identified as especially adverse genetic factors. **C.** Recursive partitioning analysis featuring clinical variables and one of any possible subsets of the adverse genetic risk factors.

Supplementary Tables

Supplementary Table 1. Number of samples and their baseline characteristics

Baseline characteristics for the whole exome analysis population, in total (n=1273) and by individual site- UAMS/UK (n=461), MMRF (n=759), and DFCI (n=53). Baseline characteristics are also presented for the prognostic analysis population (n=784) and validation population comprised of TT patients (n=85).

	Whole Exome Analysis Population (N=1273)	UAMS/UK (N=461)	MMRF (N=759)	DFCI (N=53)	Prognostic Analysis Population (N=784)	TT (N=85)
**Median Age (years) (range)**	66.0 (27.0 - 93.0)	68.0 (31.0 - 89.0)	64.0 (27.0 - 93.0)	63.0 (40.0 - 85.0)	63.0 (27.0 - 74.0)	60.4 (N=85) (30.4 - 75.2)
**Age ≥ 65 years (%)**	693/1273 (54%)	299/461 (65%)	372/759 (49%)	22/53 (42%)	352/784 (45%)	23/85 (27%)
**ISS Stage I (%)**	360/1170 (31%)	105/436 (24%)	255/734 (35%)	N/A	272/784 (35%)	24/85 (28%)
**ISS Stage II (%)**	442/1170 (38%)	169/436 (39%)	273/734 (37%)	N/A	298/784 (38%)	37/85 (44%)
**ISS Stage III (%)**	368/1170 (31%)	162/436 (37%)	206/734 (28%)	N/A	214/784 (27%)	24/85 (28%)
**IMWG Low Risk (%)**	83/1089 (8%)	14/337 (4%)	69/700 (10%)	0/52 (0%)	80/784 (10%)	11/85 (24%)
**IMWG Standard Risk (%)**	859/1089 (79%)	270/337 (80%)	537/700 (77%)	52/52 (100%)	597/784 (76%)	52/85 (61.2%)
**IMWG High Risk (%)**	147/1089 (13%)	53/337 (16%)	94/700 (13%)	0/52 (0%)	107/784 (14%)	22/85 (25.9%)
**t(4;14) (%)**	155/1273 (12%)	58/461 (13%)	93/759 (12%)	4/53 (8%)	108/784 (14%)	9/85 (11%)
**t(6;14) (%)**	14/1273 (1%)	5/461 (1%)	9/759 (1%)	0/53 (0%)	8/784 (1%)	5/85 (6%)
**t(8;14) (%)**	8/1273 (1%)	1/461 (0%)	7/759 (1%)	0/53 (0%)	5/784 (1%)	N/A
**t(11;14) (%)**	234/1273 (18%)	87/461 (19%)	146/759 (19%)	1/53 (2%)	146/784 (19%)	11/85 (13%)
**t(14;16) or t(14;20) (%)**	62/1273 (5%)	20/461 (4%)	40/759 (5%)	2/53 (4%)	42/784 (5%)	6/85 (7%)
**Median Follow-up (months) (95% CI)**	23.7 (23.5, 24.0)	25.0 (24.3, 26.2)	21.0 (20.5, 22.7)	29.7 (27.7, 31.5)	22.9 (21.2, 23.5)	88.3 (55.9, 96.6)
**Median PFS (months) (95% CI)**	29.6 (27.1, 31.2)	26.6 (23.9, 29.6)	36.6 (29.7, 46.9)	23.6 (16.0, 31.7)	31.2 (29.7, 39.1)	75.0 (53.1, NR)

Supplementary Table 2. Frequency and association of survival with bi-allelic inactivation of tumor suppressor genes

Bi-allelic events compared to wild type. Genes in commonly deleted regions were integrated for copy number and non-silent mutation. Bi-allelic events were defined as a patient with homozygous deletion, or mutation and loss of one allele. The full data set (n=784) was used for this analysis. P-values were determined by the log-rank test. Significant P-values are indicated in bold text.

Gene	Any allelic event	Bi-allelic events	Progression-free Survival (Bi-allelic inactivation vs. Wild Type)		Overall Survival (Bi-allelic inactivation vs. Wild Type)	
			**HR (95% CI)**	**P-value, R-squared**	**HR (95% CI)**	**P-value, R-squared**
CDKN2C	77/784 (10%)	6/784 (1%)	1.09 (0.27, 4.40)	P-val: 0.902, R^2 = 0.0%	2.53 (0.62, 10.25)	P-val: 0.179, R^2 = 1.3%
CYLD	175/784 (22%)	23/784 (3%)	0.72 (0.29, 1.74)	P-val: 0.460, R^2 = 0.3%	0.33 (0.05, 2.34)	P-val: 0.241, R^2 = 2.0%
DIS3	345/784 (44%)	46/784 (6%)	1.33 (0.80, 2.21)	P-val: 0.268, R^2 = 0.8%	0.82 (0.33, 2.05)	P-val: 0.673, R^2 = 0.3%
FAM46C	176/784 (22%)	37/784 (5%)	0.73 (0.37, 1.42)	P-val: 0.353, R^2 = 0.5%	1.39 (0.64, 3.01)	P-val: 0.400, R^2 = 0.7%
MAX	165/784 (21%)	24/784 (3%)	0.88 (0.45, 1.72)	P-val: 0.708, R^2 = 0.1%	0.66 (0.21, 2.07)	P-val: 0.470, R^2 = 0.6%
NFKBIA	111/784 (14%)	7/784 (1%)	0.00 (0.00, .)	P-val: 0.092, R^2 = 2.5%	0.00 (0.00, .)	P-val: 0.303, R^2 = 2.1%
RB1	346/784 (44%)	13/784 (2%)	1.55 (0.68, 3.52)	P-val: 0.290, R^2 = 0.7%	2.55 (0.92, 7.02)	P-val: 0.061, R^2 = 3.9%
TGDS	310/784 (40%)	10/784 (1%)	2.15 (0.88, 5.25)	P-val: 0.087, R^2 = 1.5%	2.35 (0.74, 7.48)	P-val: 0.136, R^2 = 2.3%
TP53	**81/784 (10%)**	**30/784 (4%)**	**2.84 (1.77, 4.55)**	**P-val: <.001, R^2 = 5.7%**	**4.62 (2.67, 8.00)**	**P-val: <.001, R^2 = 17.3%**
TRAF2	26/784 (3%)	6/784 (1%)	1.80 (0.67, 4.84)	P-val: 0.237, R^2 = 0.5%	1.08 (0.15, 7.75)	P-val: 0.938, R^2 = 0.0%
TRAF3	**142/784 (18%)**	**50/784 (6%)**	**0.41 (0.20, 0.84)**	**P-val: 0.011, R^2 = 3.5%**	0.37 (0.12, 1.16)	P-val: 0.075, R^2 = 4.0%

Supplementary Table 3. Significantly mutated genes (according to MutSigCV) and their association with survival.

HR and P-values for 26 significantly mutated genes were derived using a Cox proportional hazards model. The full data set (n=784) was used for this analysis. P-values were determined by the log-rank test. Significant P-values are indicated in bold text.

Gene	n/N (%)	Progression-free Survival		Overall Survival	
		**HR (95% CI)**	**P-value, R-squared**	**HR (95% CI)**	**P-value, R-squared**
ACTG1	26/784 (3%)	1.23 (0.67, 2.25)	P-val: 0.507, R^2 = 0.2%	1.52 (0.71, 3.26)	P-val: 0.283, R^2 = 0.9%
BRAF	62/784 (8%)	1.12 (0.73, 1.74)	P-val: 0.603, R^2 = 0.1%	1.03 (0.54, 1.98)	P-val: 0.922, R^2 = 0.0%
CDKN1B	12/784 (2%)	0.79 (0.25, 2.48)	P-val: 0.692, R^2 = 0.1%	1.04 (0.26, 4.21)	P-val: 0.957, R^2 = 0.0%
CYLD	27/784 (3%)	1.16 (0.59, 2.25)	P-val: 0.668, R^2 = 0.1%	0.55 (0.14, 2.25)	P-val: 0.402, R^2 = 0.7%
DIS3	78/784 (10%)	1.26 (0.85, 1.85)	P-val: 0.244, R^2 = 0.5%	0.76 (0.39, 1.50)	P-val: 0.431, R^2 = 0.6%
FAM46C	82/784 (10%)	0.85 (0.56, 1.31)	P-val: 0.466, R^2 = 0.2%	0.97 (0.54, 1.77)	P-val: 0.932, R^2 = 0.0%
FGFR3	29/784 (4%)	1.22 (0.69, 2.19)	P-val: 0.493, R^2 = 0.2%	0.85 (0.31, 2.30)	P-val: 0.746, R^2 = 0.1%
HIST1H1E	28/784 (4%)	1.05 (0.54, 2.04)	P-val: 0.896, R^2 = 0.0%	1.06 (0.39, 2.88)	P-val: 0.904, R^2 = 0.0%
HUWE1	48/784 (6%)	1.25 (0.78, 2.00)	P-val: 0.346, R^2 = 0.3%	0.50 (0.18, 1.36)	P-val: 0.166, R^2 = 1.9%
IRF4	17/784 (2%)	1.05 (0.49, 2.23)	P-val: 0.905, R^2 = 0.0%	1.08 (0.34, 3.39)	P-val: 0.898, R^2 = 0.0%
KRAS	174/784 (22%)	0.87 (0.64, 1.17)	P-val: 0.352, R^2 = 0.3%	0.82 (0.53, 1.29)	P-val: 0.393, R^2 = 0.6%
MAF	7/784 (1%)	1.35 (0.43, 4.20)	P-val: 0.610, R^2 = 0.1%	0.00 (0.00, .)	P-val: 0.253, R^2 = 2.2%
MAFB	7/784 (1%)	1.21 (0.30, 4.86)	P-val: 0.791, R^2 = 0.0%	2.79 (0.69, 11.30)	P-val: 0.134, R^2 = 1.3%
MAX	28/784 (4%)	0.94 (0.51, 1.73)	P-val: 0.847, R^2 = 0.0%	0.55 (0.17, 1.73)	P-val: 0.298, R^2 = 1.1%
NFKBIA	**10/784 (1%)**	**0.00 (0.00, .)**	**P-val: 0.041, R^2 = 3.2%**	0.00 (0.00, .)	P-val: 0.188, R^2 = 2.9%
NRAS	139/784 (18%)	1.06 (0.77, 1.45)	P-val: 0.736, R^2 = 0.0%	0.77 (0.46, 1.29)	P-val: 0.317, R^2 = 0.9%
PRKD2	30/784 (4%)	1.30 (0.73, 2.32)	P-val: 0.379, R^2 = 0.3%	0.98 (0.36, 2.65)	P-val: 0.961, R^2 = 0.0%
PTPN11	20/784 (3%)	0.96 (0.45, 2.03)	P-val: 0.903, R^2 = 0.0%	1.27 (0.47, 3.46)	P-val: 0.633, R^2 = 0.2%
RASA2	9/784 (1%)	0.86 (0.27, 2.68)	P-val: 0.791, R^2 = 0.0%	1.33 (0.33, 5.38)	P-val: 0.689, R^2 = 0.1%
RB1	15/784 (2%)	1.60 (0.75, 3.39)	P-val: 0.216, R^2 = 0.5%	2.07 (0.76, 5.60)	P-val: 0.146, R^2 = 1.4%
SP140	20/784 (3%)	1.10 (0.49, 2.48)	P-val: 0.814, R^2 = 0.0%	1.74 (0.71, 4.27)	P-val: 0.218, R^2 = 1.1%
TGDS	**9/784 (1%)**	**2.61 (1.07, 6.34)**	**P-val: 0.028, R^2 = 1.3%**	2.55 (0.81, 8.04)	P-val: 0.097, R^2 = 1.6%
TP53	**45/784 (6%)**	**2.27 (1.49, 3.46)**	**P-val: <.001, R^2 = 4.5%**	**3.58 (2.16, 5.94)**	**P-val: <.001, R^2 = 14.4%**
TRAF2	16/784 (2%)	1.47 (0.73, 2.97)	P-val: 0.283, R^2 = 0.4%	1.72 (0.63, 4.66)	P-val: 0.281, R^2 = 0.8%
TRAF3	**48/784 (6%)**	**0.45 (0.22, 0.92)**	**P-val: 0.024, R^2 = 2.4%**	**0.25 (0.06, 1.02)**	**P-val: 0.036, R^2 = 5.1%**
UBR5	24/784 (3%)	0.87 (0.41, 1.84)	P-val: 0.707, R^2 = 0.1%	1.08 (0.40, 2.92)	P-val: 0.883, R^2 = 0.0%

Supplementary Table 4. Recurrent copy number abnormalities and their association with survival.

Hazard ratios and P-values for recurrent copy number abnormalities significantly associated with PFS and OS were derived using Cox proportional hazards models. The data set used n=784. P-values were determined by the log-rank test. Significant P-values are indicated in bold text.

Gene	Gain/ Loss	Cytoband	n/N (%)	Progression-free Survival		Overall Survival	
				HR (95% CI)	P-value, R-squared	HR (95% CI)	P-value, R-squared
CKS1B	**Gain**	**1q21.3**	**226/784 (29%)**	**1.53 (1.19, 1.98)**	**P-val: 0.001, R^2 = 3.9%**	**1.80 (1.25, 2.60)**	**P-val: 0.002, R^2 = 7.6%**
MYC	Gain	8q24.2	61/784 (8%)	1.08 (0.69, 1.71)	P-val: 0.729, R^2 = 0.0%	1.73 (0.99, 3.02)	P-val: 0.052, R^2 = 2.7%
CCND1	Gain	11q13.3	304/784 (39%)	0.80 (0.62, 1.04)	P-val: 0.093, R^2 = 1.1%	0.88 (0.61, 1.29)	P-val: 0.520, R^2 = 0.4%
MAF	Gain	16q23.2	11/784 (1%)	1.74 (0.78, 3.92)	P-val: 0.173, R^2 = 0.6%	2.31 (0.85, 6.27)	P-val: 0.090, R^2 = 1.8%
AKAP1	Gain	17q22	69/784 (9%)	0.91 (0.58, 1.44)	P-val: 0.693, R^2 = 0.1%	1.13 (0.61, 2.11)	P-val: 0.696, R^2 = 0.1%
CRBN	Gain	3p	267/784 (34%)	0.81 (0.63, 1.06)	P-val: 0.127, R^2 = 0.9%	0.81 (0.55, 1.20)	P-val: 0.292, R^2 = 1.0%
ADCY2	Gain	5p	321/784 (41%)	0.85 (0.66, 1.09)	P-val: 0.194, R^2 = 0.7%	1.05 (0.73, 1.51)	P-val: 0.796, R^2 = 0.1%
TNFAIP8	Gain	5q	307/784 (39%)	0.80 (0.61, 1.03)	P-val: 0.080, R^2 = 1.2%	0.92 (0.63, 1.34)	P-val: 0.661, R^2 = 0.2%
TNXB	Gain	6p	140/784 (18%)	0.77 (0.55, 1.09)	P-val: 0.141, R^2 = 0.9%	1.06 (0.67, 1.69)	P-val: 0.803, R^2 = 0.1%
RAPGEF5	Gain	7p	244/784 (31%)	0.80 (0.61, 1.05)	P-val: 0.105, R^2 = 1.1%	0.97 (0.66, 1.44)	P-val: 0.879, R^2 = 0.0%
KLF14	**Gain**	**7q**	**246/784 (31%)**	**0.74 (0.56, 0.98)**	**P-val: 0.035, R^2 = 1.8%**	0.86 (0.57, 1.28)	P-val: 0.452, R^2 = 0.5%
RNF20	Gain	9q	403/784 (51%)	0.78 (0.61, 1.00)	P-val: 0.053, R^2 = 1.5%	0.89 (0.62, 1.28)	P-val: 0.531, R^2 = 0.3%
RRAS2	Gain	11p	253/784 (32%)	0.77 (0.59, 1.01)	P-val: 0.059, R^2 = 1.4%	0.98 (0.67, 1.45)	P-val: 0.937, R^2 = 0.0%
BLM	**Gain**	**15q**	**370/784 (47%)**	**0.78 (0.61, 1.00)**	**P-val: 0.049, R^2 = 1.5%**	0.91 (0.63, 1.30)	P-val: 0.600, R^2 = 0.2%
WDR72	Gain	15q	372/784 (47%)	0.82 (0.64, 1.05)	P-val: 0.117, R^2 = 1.0%	0.88 (0.61, 1.26)	P-val: 0.472, R^2 = 0.4%
ZNF426	Gain	19p	405/784 (52%)	0.82 (0.64, 1.05)	P-val: 0.116, R^2 = 1.0%	1.06 (0.74, 1.53)	P-val: 0.733, R^2 = 0.1%
ZNF227	**Gain**	**19q**	**358/784 (46%)**	**0.76 (0.59, 0.98)**	**P-val: 0.031, R^2 = 1.8%**	0.86 (0.60, 1.24)	P-val: 0.426, R^2 = 0.5%
CHODL	Gain	21q	176/784 (22%)	0.85 (0.63, 1.15)	P-val: 0.280, R^2 = 0.5%	1.02 (0.66, 1.57)	P-val: 0.923, R^2 = 0.0%
SON	Gain	21q	190/784 (24%)	0.81 (0.60, 1.09)	P-val: 0.165, R^2 = 0.8%	1.06 (0.70, 1.60)	P-val: 0.792, R^2 = 0.1%
FAM46C	**Loss**	**1p12**	**116/784 (15%)**	1.33 (0.96, 1.84)	P-val: 0.089, R^2 = 1.0%	**2.04 (1.34, 3.13)**	**P-val: <.001, R^2 = 7.8%**
RPL5	**Loss**	**1p22.1**	**153/784 (20%)**	1.22 (0.90, 1.66)	P-val: 0.197, R^2 = 0.6%	**1.74 (1.16, 2.61)**	**P-val: 0.007, R^2 = 5.3%**
CDKN2C	**Loss**	**1p32.3**	**74/784 (9%)**	1.27 (0.84, 1.94)	P-val: 0.259, R^2 = 0.5%	**2.35 (1.43, 3.84)**	**P-val: <.001, R^2 = 7.7%**
DNMT3A	Loss	2p23.3	24/784 (3%)	1.60 (0.90, 2.86)	P-val: 0.108, R^2 = 0.9%	1.88 (0.88, 4.04)	P-val: 0.099, R^2 = 1.8%
FGFR3	**Loss**	**4p16.3**	**64/784 (8%)**	1.20 (0.75, 1.92)	P-val: 0.439, R^2 = 0.2%	**1.93 (1.10, 3.38)**	**P-val: 0.019, R^2 = 3.7%**
PARK2	**Loss**	**6q26**	**119/784 (15%)**	1.30 (0.94, 1.81)	P-val: 0.108, R^2 = 0.9%	**1.81 (1.17, 2.79)**	**P-val: 0.007, R^2 = 5.2%**
CDKN2A	Loss	9p21.3	22/784 (3%)	0.97 (0.46, 2.06)	P-val: 0.942, R^2 = 0.0%	1.27 (0.47, 3.44)	P-val: 0.638, R^2 = 0.2%
TRAF2	**Loss**	**9q34.3**	**14/784 (2%)**	**2.03 (1.00, 4.11)**	**P-val: 0.044, R^2 = 1.2%**	0.95 (0.23, 3.82)	P-val: 0.935, R^2 = 0.0%
BIRC3	Loss	11q22.1	20/784 (3%)	1.37 (0.68, 2.78)	P-val: 0.376, R^2 = 0.3%	1.36 (0.50, 3.68)	P-val: 0.548, R^2 = 0.3%
ATM	Loss	11q22.3	19/784 (2%)	1.36 (0.64, 2.88)	P-val: 0.422, R^2 = 0.2%	1.53 (0.57, 4.16)	P-val: 0.396, R^2 = 0.5%
CDKN1B	Loss	12p13.1	71/784 (9%)	1.40 (0.95, 2.07)	P-val: 0.084, R^2 = 1.1%	1.67 (0.99, 2.83)	P-val: 0.053, R^2 = 2.7%
BRCA2	Loss	13q13.1	331/784 (42%)	1.24 (0.97, 1.58)	P-val: 0.092, R^2 = 1.1%	1.24 (0.86, 1.78)	P-val: 0.251, R^2 = 1.1%
RB1	Loss	13q14.2	343/784 (44%)	1.27 (0.99, 1.62)	P-val: 0.060, R^2 = 1.4%	1.35 (0.94, 1.93)	P-val: 0.104, R^2 = 2.2%
DIS3	Loss	13q21.33	317/784 (40%)	1.23 (0.96, 1.57)	P-val: 0.107, R^2 = 1.0%	1.32 (0.92, 1.89)	P-val: 0.137, R^2 = 1.8%
ABCD4	Loss	14q24.3	188/784 (24%)	1.09 (0.82, 1.46)	P-val: 0.542, R^2 = 0.1%	1.11 (0.73, 1.70)	P-val: 0.611, R^2 = 0.2%
TRAF3	Loss	14q32.32	129/784 (16%)	0.84 (0.59, 1.20)	P-val: 0.337, R^2 = 0.4%	1.08 (0.67, 1.75)	P-val: 0.745, R^2 = 0.1%
CYLD	Loss	16q12.1	166/784 (21%)	0.88 (0.64, 1.20)	P-val: 0.407, R^2 = 0.3%	1.11 (0.72, 1.72)	P-val: 0.626, R^2 = 0.2%
WWOX	Loss	16q23.1	191/784 (24%)	1.11 (0.84, 1.48)	P-val: 0.461, R^2 = 0.2%	1.43 (0.96, 2.12)	P-val: 0.076, R^2 = 2.5%
TP53	**Loss**	**17p13.1**	**63/784 (8%)**	1.39 (0.92, 2.10)	P-val: 0.120, R^2 = 0.9%	**2.43 (1.49, 3.98)**	**P-val: <.001, R^2 = 8.3%**
DOCK5	Loss	8p	150/784 (19%)	1.01 (0.74, 1.38)	P-val: 0.949, R^2 = 0.0%	1.19 (0.76, 1.85)	P-val: 0.442, R^2 = 0.5%

Supplementary Table 5. The association of chromosomal translocations and copy number clusters with survival.

The hazard ratios and P-values for the association of translocations with PFS and OS were derived using Cox proportional hazards models using the n=784 dataset. Each translocation was compared to all patients either without a translocation or with a different translocation. Each copy number cluster was compared against cluster 2. P-values were determined by the log-rank test. Significant P-values are indicated in bold text. NS = not significant. NA = Not Available.

Feature	n/N (%)	Progression-free Survival		Overall Survival	
		HR (95% CI)	P-value, R-squared	HR (95% CI)	P-value, R-squared
**t(4;14)**	**108/784 (14%)**	**1.58 (1.15, 2.17)**	**P-val: 0.004, R^2 = 2.8%**	1.32 (0.82, 2.14)	P-val: 0.254, R^2 = 1.0%
**t(6;14)**	8/784 (1%)	1.19 (0.38, 3.73)	P-val: 0.761, R^2 = 0.0%	1.98 (0.49, 8.01)	P-val: 0.331, R^2 = 0.6%
**t(8;14)**	5/784 (1%)	0.66 (0.09, 4.69)	P-val: 0.673, R^2 = 0.1%	0.00 (0.00, .)	P-val: 0.416, R^2 = 1.1%
**t(11;14)**	146/784 (19%)	1.05 (0.77, 1.44)	P-val: 0.763, R^2 = 0.0%	0.71 (0.43, 1.19)	P-val: 0.192, R^2 = 1.5%
**t(14;16) or t(14;20)**	42/784 (5%)	1.09 (0.65, 1.84)	P-val: 0.736, R^2 = 0.0%	1.77 (0.95, 3.29)	P-val: 0.068, R^2 = 2.3%
**MYC Translocation**	202/751 (27%)	1.05 (0.80, 1.39)	P-val: 0.723, R^2 = 0.1%	1.32 (0.89, 1.94)	P-val: 0.164, R^2 = 1.6%
**APOBEC Signature**	**30/784 (4%)**	**1.13 (0.62, 2.07)**	P-val: 0.685, R^2 = 0.1%	**2.01 (1.02, 3.97)**	**P-val: 0.040, R^2 = 2.8%**
**Hyperdiploid**	453/784 (58%)	0.86 (0.67, 1.10)	P-val: 0.220, R^2 = 0.6%	1.01 (0.70, 1.46)	P-val: 0.945, R^2 = 0.0%
**LOH Percent > 4.6**	**48/784 (6%)**	**2.11 (1.37, 3.24)**	**P-val: <.001, R^2 = 3.6%**	**2.52 (1.44, 4.42)**	**P-val: <.001, R^2 = 6.8%**
**Homologous recombination deficiency mutations**	78/784 (10%)	0.79 (0.50, 1.25)	P-val: 0.319, R^2 = 0.4%	0.87 (0.45, 1.66)	P-val: 0.666, R^2 = 0.2%
**CN-Cluster 1 (vs 2)**	110/364 (30%)	1.25 (0.84, 1.87)	P-val: 0.277, R^2 = 1.0%	1.21 (0.68, 2.15)	P-val: 0.521, R^2 = 0.7%
**CN-Cluster 3 (vs 2)**	44/298 (15%)	1.18 (0.64, 2.18)	P-val: 0.592, R^2 = 0.3%	1.86 (0.92, 3.76)	P-val: 0.079, R^2 = 5.5%
**CN-Cluster 4 (vs 2)**	39/293 (13%)	1.48 (0.85, 2.57)	P-val: 0.167, R^2 = 1.9%	1.33 (0.59, 2.99)	P-val: 0.488, R^2 = 1.0%
**CN-Cluster 5 (vs 2)**	57/311 (18%)	1.07 (0.63, 1.81)	P-val: 0.804, R^2 = 0.1%	0.97 (0.45, 2.08)	P-val: 0.935, R^2 = 0.0%
**CN-Cluster 6 (vs 2)**	47/301 (16%)	0.92 (0.50, 1.71)	P-val: 0.802, R^2 = 0.1%	0.92 (0.39, 2.18)	P-val: 0.845, R^2 = 0.1%
**CN-Cluster 7 (vs 2)**	**67/321 (21%)**	**1.94 (1.29, 2.91)**	**P-val: 0.001, R^2 = 8.2%**	1.36 (0.73, 2.52)	P-val: 0.325, R^2 = 1.8%
**CN-Cluster 8 (vs 2)**	125/379 (33%)	1.27 (0.87, 1.87)	P-val: 0.217, R^2 = 1.3%	0.71 (0.37, 1.35)	P-val: 0.292, R^2 = 2.3%
**CN-Cluster 9 (vs 2)**	41/295 (14%)	1.36 (0.76, 2.40)	P-val: 0.297, R^2 = 1.1%	1.22 (0.54, 2.73)	P-val: 0.633, R^2 = 0.5%

Supplementary Table 6. The association of selected interactions with survival by univariate Cox analysis.

The P-values for the association of interactions with PFS and OS were derived using Cox proportional hazards models based on the analysis dataset (n=784). Each interaction was tested in a model featuring only the main effects and interaction term. Interaction terms tested but not shown below include: CKS1B*Non-HRD, CKS1B*ABCD4 Loss (borderline-significant interaction for PFS, P-value = 0.0538), CKS1B*CDKN1B Loss, MYC translocation*FAM46C Loss, ZNF426 Gain*FAM46C Loss (significant interaction for PFS, P-value = 0.0202), and t(11;14)*Any Loss of 11q. Significant P-values are indicated in bold text.

Gene Interaction	Term	P-value (PFS)	P-value (OS)
t(4;14) * Bi-allelic *TP53*	t(4;14)	**0.0418**	0.7691
	Bi-allelic *TP53* (three-level)	**<0.0001**	**<0.0001**
	Interaction	**0.0217**	0.1004
CKS1B * Bi-allelic *TP53*	*CKS1B* (three-level)	**0.0003**	**<0.0001**
	Bi-allelic *TP53*	**<0.0001**	**<0.0001**
	Interaction	0.6491	0.5142
CKS1B * t(4;14)	*CKS1B* (three-level)	**0.0293**	**0.0034**
	t(4;14)	0.5004	0.8303
	Interaction	0.5487	0.9655
CKS1B * *FAM46C* Loss	*CKS1B* (three-level)	**0.0259**	0.2294
	FAM46C Loss	0.8211	0.6228
	Interaction	0.1994	**0.0461**
CKS1B * t(14:16)/t(14:20)	*CKS1B* (three-level)	**0.0004**	**0.0030**
	t(14:16)/t(16:20)	0.8023	0.4509
	Interaction	0.8842	0.8796
CKS1B * t(14:16)	*CKS1B* (three-level)	**0.0002**	**0.0011**
	t(14:16)	0.6066	0.6120
	Interaction	0.5404	0.9029
CKS1B * del17p	*CKS1B* (three-level)	**0.0002**	**<0.0001**
	del17p	0.0854	**<0.0001**
	Interaction	0.8280	0.4751

P-values determined from Wald chi-square joint tests.

Supplementary Table 7. Univariate PFS associations and hazard ratios.
Tabulated data to go with Figure 3.

Progression Free Survival				
**Variable**	**n/N (%)**	**HR (95% CI)**	**P-value**	**R^2**
ISS Stage (II vs I)	298/570 (52%)	1.68 (1.20, 2.34)	0.002	6.3%
ISS Stage (III vs I)	214/486 (44%)	3.13 (2.27, 4.32)	<0.001	26.9%
Age ≥ 65 years	352/784 (45%)	1.69 (1.32, 2.17)	<0.001	6.6%
t(4;14)	108/784 (14%)	1.58 (1.15, 2.17)	0.004	2.8%
LOH >4.6%	48/784 (6%)	2.11 (1.37, 3.24)	<0.001	3.6%
CN-Cluster 7 (vs 2)	67/321 (21%)	1.94 (1.29, 2.91)	0.001	8.2%
CKS1B (1q21.3) (Gain vs. Normal)	173/731 (24%)	1.36 (1.01, 1.81)	0.040	1.7%
CKS1B (1q21.3) (Amplification vs. Normal)	53/611 (9%)	2.16 (1.45, 3.23)	<0.001	5.9%
Amplification MYC (8q24)	6/784 (1%)	3.13 (1.16, 8.42)	0.017	1.4%
TRAF2 (9q34.3) (Wild Type vs. Abnormal)	26/784 (3%)	1.74 (1.01, 2.98)	0.042	1.3%
RB1 (13q14.2) (Wild Type vs. Abnormal)	346/784 (44%)	1.28 (1.00, 1.64)	0.048	1.5%
Any loss chr13q	354/784 (45%)	1.33 (1.04, 1.70)	0.024	2.0%
TP53 (17p13.1) (Bi-allelic vs. Wild Type)	30/733 (4%)	2.84 (1.77, 4.55)	<0.001	5.7%
Gain/Amplification 7q (*KLF14*)	246/784 (31%)	0.74 (0.56, 0.98)	0.035	1.8%
Gain/Amplification 15q (*BLM*)	370/784 (47%)	0.78 (0.61, 1.00)	0.049	1.5%
Gain/Amplification 19q (*ZNF227*)	358/784 (46%)	0.76 (0.59, 0.98)	0.031	1.8%
Mutated *TGDS*	9/784 (1%)	2.61 (1.07, 6.34)	0.028	1.3%
Mutated *TRAF3*	48/784 (6%)	0.45 (0.22, 0.92)	0.024	2.4%

Supplementary Table 8. Univariate OS associations and hazard ratios.
Tabulated data to go with Figure 3.

Overall Survival				
**Variable**	**n/N (%)**	**HR (95% CI)**	**P-value**	**R^2**
ISS Stage (II vs I)	298/570 (52%)	2.46 (1.42, 4.26)	<0.001	16.8%
ISS Stage (III vs I)	214/486 (44%)	4.54 (2.67, 7.72)	<0.001	39.7%
Age ≥ 65 years	352/784 (45%)	1.66 (1.15, 2.38)	0.006	6.1%
APOBEC Signature	30/784 (4%)	2.01 (1.02, 3.97)	0.040	2.8%
LOH >4.6%	48/784 (6%)	2.52 (1.44, 4.42)	<0.001	6.8%
CN-Cluster 3 (vs 2)	44/298 (15%)	1.86 (0.92, 3.76)	0.079	5.5%
CDKN2C (1p32.3) (One Allele vs. Wild Type)	71/778 (9%)	2.13 (1.27, 3.57)	0.003	5.8%
CDKN2C (1p32.3) (Abnormal vs. Wild Type)	77/784 (10%)	2.18 (1.33, 3.56)	0.001	6.6%
Loss *FAM46C* (1p12)	116/784 (15%)	2.04 (1.34, 3.13)	<0.001	7.8%
Loss *RPL5* (1p22.1)	153/784 (20%)	1.74 (1.16, 2.61)	0.007	5.3%
CKS1B (1q21.3) (Gain vs. Normal)	173/731 (24%)	1.50 (0.98, 2.30)	0.057	3.3%
CKS1B (1q21.3) (Amplification vs. Normal)	53/611 (9%)	2.77 (1.63, 4.71)	<0.001	12.3%
Loss *FGFR3* (4p16.3)	64/784 (8%)	1.93 (1.10, 3.38)	0.019	3.7%
Loss *PARK2* (6q26)	119/784 (15%)	1.81 (1.17, 2.79)	0.007	5.2%
TP53 (17p13.1) (Bi-allelic vs. Wild Type)	30/733 (4%)	4.62 (2.67, 8.00)	<0.001	17.3%
TP53 (17p13.1) (Abnormal vs. Wild Type)	81/784 (10%)	2.34 (1.48, 3.70)	<0.001	9.0%
Mutated *TRAF3*	48/784 (6%)	0.25 (0.06, 1.02)	0.036	5.1%

Supplementary Table 9. Multivariate PFS associations and hazard ratios.
Tabulated data to go with Figure 3.

Progression Free Survival				
**Variable**	**n/N (%)**	**HR (95% CI)**	**P-value**	**Cumulative R^2**
TP53 (Bi-allelic vs. Wild Type)	30/733 (4%)	2.419 (1.38, 3.95)	0.0009	/
TP53 (One allele inactive vs. Wild Type)	51/754 (7%)	0.466 (0.206, 0.914)	0.0423	5.8
1q21.3 (*CKS1B*) (Gain vs. Normal)	173/731 (24%)	1.173 (0.873, 1.576)	0.2905	/
1q21.3 (*CKS1B*) (Amplification vs. Normal)	53/611 (9%)	1.952 (1.291, 2.952)	0.0015	10.7
LOH >4.6%	48/784 (6%)	1.938 (1.223, 3.071)	0.0049	13.9
t(4;14)	108/784 (14%)	1.383 (0.953, 1.961)	0.0773	15.8
t(4;14) & *TP53* (One allele vs. Wild Type)	7/108 (6%)	7.658 (2.282, 23.33)	0.0046	/
t(4;14) & *TP53* (Bi-allelic vs. Wild Type)	5/108 (5%)	2.089 (0.481, 6.381)	0.5322	18.4
ISS Stage (II vs I)	298/570 (52%)	1.500 (1.071, 2.101)	0.0182	/
ISS Stage (III vs I)	214/486 (44%)	2.671 (1.926, 3.702)	<0.0001	30.8
Age ≥ 65 years	352/784 (45%)	1.599 (1.240, 2.063)	0.0003	34.3

Supplementary Table 10. Multivariate OS associations and hazard ratios. Tabulated data to go with Figure 3.

Overall Survival				
**Variable**	**n/N (%)**	**HR (95% CI)**	**P-value**	**Cumulative R^2**
TP53 (Bi-allelic vs. Wild Type)	30/733 (4%)	4.574 (2.636, 7.936)	<0.0001	/
TP53 (One allele inactive vs. Wild Type)	51/754 (7%)	1.291 (0.624, 2.674)	0.4911	16.5
1q21.3 (*CKS1B*) (Gain vs. Normal)	173/731 (24%)	1.395 (0.912, 2.134)	0.1244	/
1q21.3 (*CKS1B*) (Amplification vs. Normal)	53/611 (9%)	2.619 (1.534, 4.472)	0.0004	25.2
ISS Stage (II vs I)	298/570 (52%)	2.389 (1.377, 4.144)	0.002	/
ISS Stage (III vs I)	214/486 (44%)	4.258 (2.498, 7.258)	<0.0001	44.5
Age ≥ 65 years	352/784 (45%)	1.474 (1.020, 2.131)	0.0391	46.5

Supplementary Table 11. Comparison of patients being low, intermediate, Double Hit Risk in the recursive partitioning model by either ISS or IMWG in the MGP patient set.

A total of 784 MGP patients used for recursive partitioning analyses had data available to compute ISS Stage and IMWG risk classification.

	ISS I	ISS II	ISS III	Total
MGP Recursive Partitioning Low-Risk	248 (31.6%)	139 (17.7%)	0 (0%)	387 (49.4%)
MGP Recursive Partitioning Intermediate-Risk	14 (1.8%)	148 (18.9%)	187 (23.9%)	349 (44.5%)
MGP Recursive Partitioning Double-Hit	10 (1.3%)	11 (1.4%)	27 (3.4%)	48 (6.1%)
Total	272 (34.7%)	298 (38.0%)	214 (27.3%)	784 (100%)

	IMWG Low-Risk	IMWG Standard-Risk	IMWG High-Risk	Total
MGP Recursive Partitioning Low-Risk	80 (10.2%)	277 (35.3%)	30 (3.8%)	387 (49.4%)
MGP Recursive Partitioning Intermediate-Risk	0 (0%)	296 (37.8%)	53 (6.8%)	349 (44.5%)
MGP Recursive Partitioning Double-Hit	0 (0%)	24 (3.1%)	24 (3.1%)	48 (6.1%)
Total	80 (10.2%)	597 (76.1%)	107 (13.6%)	784 (100%)

Supplementary Table 12. Indicators used for chromosomes featuring multiple genes with extremely high concordance in copy number data.

Indicator	Genes considered for indicator	Concordance
**Gain 5**	*ADCY2, TNFAIP8*	0.949
**Gain 7**	*RAPGEF5, KLF14*	0.954
**Gain 15**	*BLM, WDR72*	0.964
**Gain 19**	*ZNF426, ZNF227*	0.899
**Gain 21**	*CHODL, SON*	0.964
**Loss 11q**	*BIRC3, ATM*	0.986
**Loss 13q**	*BRCA2, RB1, DIS3*	0.967 (BRCA2 vs. RB1)<br>0.936 (BRCA2 vs. DIS3)<br>0.954 (RB1 vs. DIS3)
